# Plac1 Is a Key Regulator of the Inflammatory Response and Immune Tolerance In Mammary Tumorigenesis

**DOI:** 10.1038/s41598-018-24022-w

**Published:** 2018-04-09

**Authors:** Hongyan Yuan, Xiaoyi Wang, Chunmei Shi, Lu Jin, Jianxia Hu, Alston Zhang, James Li, Nairuthya Vijayendra, Venkata Doodala, Spencer Weiss, Yong Tang, Louis M. Weiner, Robert I. Glazer

**Affiliations:** 1 Department of Oncology and Lombardi Comprehensive Cancer Center, Georgetown University, Washington, DC 20007 USA; 2 Laboratory of Thyroid Diseases, the Affiliated Hospital of Qingdao University, Qingdao, 266003 China; 3 Department of Bioinformatics, Biostatistics and Biomathematics, Georgetown University Medical Center, Washington, DC 20007 USA

## Abstract

Plac1 is an X-linked trophoblast gene expressed at high levels in the placenta, but not in adult somatic tissues other than the testis. Plac1 however is re-expressed in several solid tumors and in most human cancer cell lines. To explore the role of Plac1 in cancer progression, Plac1 was reduced by RNA interference in EO771 mammary carcinoma cells. EO771 “knockdown” (KD) resulted in 50% reduction in proliferation *in vitro* and impaired tumor growth in syngeneic mice; however, tumor growth in SCID mice was equivalent to tumor cells expressing a non-silencing control RNA, suggesting that Plac1 regulated adaptive immunity. Gene expression profiling of Plac1 KD cells indicated reduction in several inflammatory and immune factors, including Cxcl1, Ccl5, Ly6a/Sca-1, Ly6c and Lif. Treatment of mice engrafted with wild-type EO771 cells with a Cxcr2 antagonist impaired tumor growth, reduced myeloid-derived suppressor cells and regulatory T cells, while increasing macrophages, dendritic cells, NK cells and the penetration of CD8+ T cells into the tumor bed. Cxcl1 KD phenocopied the effects of Plac1 KD on tumor growth, and overexpression of Cxcl1 partially rescued Plac1 KD cells. These results reveal that Plac1 modulates a tolerogenic tumor microenvironment in part by modulating the chemokine axis.

## Introduction

Placental-specific protein 1 (Plac1) is an Xq26-linked gene that encodes a microvillous membrane protein expressed primarily in trophoblasts, at low levels in the testis, but not in other adult somatic tissues^1^, and has the most restricted normal tissue expression pattern in comparison to other cancer/testis antigens^2^. Silva first reported that Plac1 RNA was expressed over a 4-log range in >50% of human cancer cell lines covering 17 different malignancies^2^, suggesting that some cancers mirror an onco-placental disease or a “somatic cell pregnancy”^3^. This hypothesis has been confirmed by the detection of Plac1 in malignancies of the breast^4–6^, endometrium^7^, ovary^7^, lung^2,8^, liver^9^, colon^6,10,11^, stomach^12^ and prostate^13^. In colorectal cancer biopsies, higher levels of Plac1 were detected in 50% of stage III/IV disease in comparison to early stage disease^9,10^, and Plac1-dependent cytotoxic T cell (CTL) activity correlated with overall survival^11^.

In the MMTV-PPARd transgenic model of luminal B breast cancer, Plac1 expression was highly elevated at the onset and throughout mammary tumorigenesis^14^, suggesting that it might have a role in the initiation and progression of tumor development. Previous studies found that Plac1 transcription in human breast cancer cells was regulated by many of the same co-activators associated with PPARd and other nuclear receptors^15–17^, including C/EBPβ and NCOA3^18,19^, both of which have been implicated in breast cancer progression^16,20–22^. Despite these findings, little is known about the oncogenic processes downstream of Plac1. To address this question, EO771 mammary carcinoma cells, which express high levels of Plac1, were used to examine gene expression and signaling pathways under the control of Plac1. Our findings reveal that Plac1 regulates a chemokine and immune tolerogenic signaling network necessary for sustaining tumor growth, which suggests potential therapeutic strategies that could alter the tumor microenvironment to make it more amenable to therapy.

## Results

### Reduction of Plac1 inhibits EO771 cell growth and tumor formation

**Figure 1:**
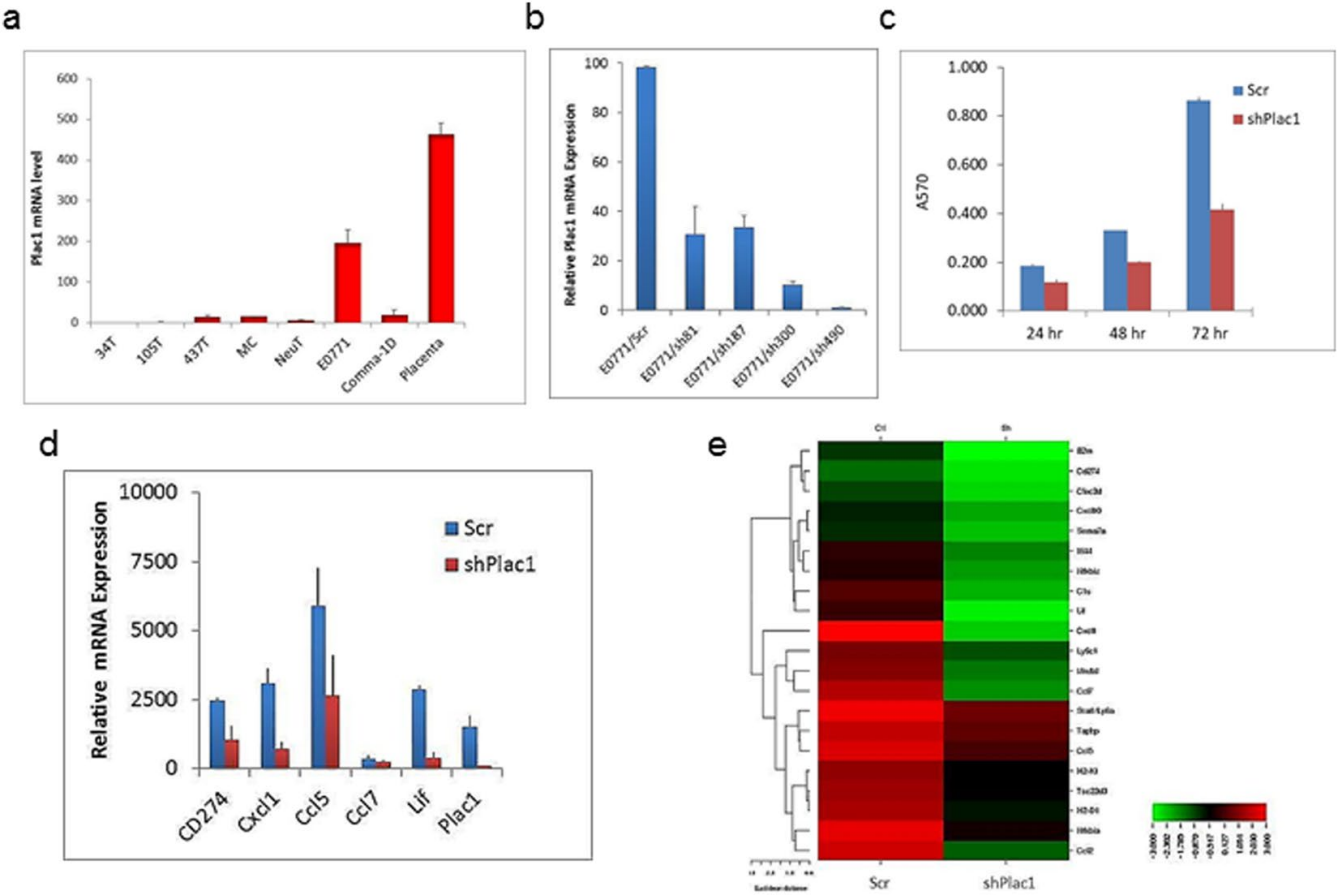
Plac1 expression and lentivirus-mediated reduction of Plac1 in EO771 cells. (**a**) EO771 mouse mammary tumor cells expressed high levels of Plac1 in comparison to mouse placenta. (**b**) EO771 cells were transduced with lentiviruses expressing crambled RNA (Scr) or four Plac1 shRNAs designated sh81, sh187, sh300 and sh490; sh490 inhibited RNA expression >98%, and these cells were designated EO771/shPlac1. (**c**) EO771/Scr and EO771/shPlac1 cells were grown as monolayers, and the number of viable cells were quantified by sulforhodamine B staining. Shown is the mean ± S.D. of triplicate analysis of three samples. The growth of EO771/shPlac1 cells differed significantly (*P* < 0.001) from EO771/Scr cells by the two-sided Student’s t test. (**d**) qRT-PCR analysis of immune cell-related gene expression downregulated in EO771/shPlac1 cells. Shown is the mean ± S.D. of triplicate analysis of three samples. Significant differences between EO771/Scr and EO771/shPlac1 cells were obtained for CD274 (*P* < 0.01), Plac1 (*P* < 0.01), Cxcl1 (*P* < 0.001), Ccl5 (*P* < 0.001) and Lif (*P* < 0.001) using the two-tailed Student’s t test; values for Ccl7 were not significantly different (*P* > 0.05). (**e**) Heatmap of gene expression as determined by Affymetrix microarray analysis of EO771/Scr (Ctl) and EO771/shPlac1 (sh) cells. Shown are immune cell-related transcripts (Table 1) representing ≥3.0-fold change in expression.

To characterize the functional role of Plac1, several mouse mammary tumor cell lines were screened by qRT-PCR for Plac1 RNA expression; among these, EO771 cells expressed the highest level, which was substantial in comparison to mouse placenta (Fig. 1a). EO771 cells were then transduced with recombinant lentiviruses expressing shRNAs targeting four regions of Plac1 mRNA (Fig. 1b). shRNA490 produced >98% reduction of Plac1 expression, and EO771 cells transduced with this shRNA (EO771/shPlac1) were used for further studies. EO771/shPlac1 cells grew in monolayer culture at 50% of the rate of control cells expressing a non-silencing RNA (Fig. 1c). Gene expression profiling revealed that Plac1 markedly suppressed several chemokine genes, including Cxcl1, Ccl7, Ccl2, Ccl5 and Cxcl10, as well as immune-related factors Lif, Ly6a/Sca-1, Ly6c and CD274 (Table 1, Fig. 1d, Supplementary Table 2). Changes in the expression of several of these genes were confirmed by qRT-PCR and most were consistent with the array profile (Fig. 1e).

**Table 1:** Expression of immune-related genes in E0771/shPlac1 cells. Shown are ≥3.0-fold changes in expression with a raw score ≥300 in either E0771/shPlac1 or E0771/Scr cells.

Gene	Raw Score		shPlac1/Scr	Function
	Scr	shPlac1		
Cxcl1	7054	106	−67	Cxcr2 ligand
Ccl7	2854	153	−19	Ccr3 ligand
CD68	931	55	−18	Macrophage phagocytosis
Ccl2	3640	305	−12	Ccr2/Ccr5 ligand; MDSC
Lif	664	80	−8.3	Immune tolerance at maternal−fetal interface
C1	918	128	−7.1	Complement
Ccl5	3812	784	−4.9	Ccr1/3/4/5 ligand
Tsc22d3	2265	478	−4.8	Mediates IL10 immunosuppression
Ly6a	4937	1160	−4.3	Sca-1; inhibits TGFb, Pten and PPARg
Ly6c	1198	329	−3.7	Mono/Mφ marker; MDSC
Clec2d	366	99	−3.7	Protects against NK cells
Cxcl10	459	130	−3.6	Cxcr3 ligand
CD274	302	91	−3.4	PD-L1; PD-1 ligand

### Plac1 reduction blocks tumor growth in syngeneic mice, but not in SCID mice

**Figure 2:**
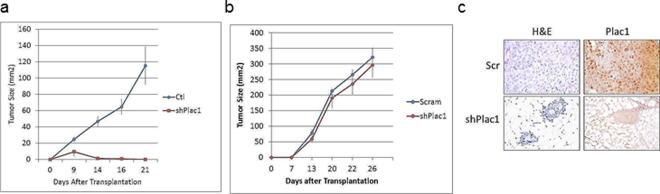
Growth of EO771/Scr and EO771/shPlac1 cells in syngeneic and SCID mice. (**a**) Syngeneic C57BL/6 mice or (**b**) SCID mice at five weeks of age, were inoculated in the mammary gland with 1 × 10^6^ cells. Tumor size was measured by calipers in two dimensions. Tumor growth for EO771/Scr and EO771/shPlac1 cells in syngeneic mice differed significantly (P = 0.040) by the unpaired Student’s t test. There was no significant difference (P > 0.05) in tumor growth between the two cell lines in SCID mice. Shown is the mean ± SD, N = 5 per group. (**c**) H&E staining and Plac1 IHC in isografts of EO771/Scr and EO771/shPlac1 cells. Magnification 400X.

To determine the influence of Plac1 on tumor development in a host, EO771/shPlac1 cells were implanted in syngeneic C57BL/6 mice, and growth monitored by caliper measurement (Fig. 2a). EO771/shPlac1 isografts grew transiently in syngeneic mice, but when transplanted into SCID mice, their growth was similar to EO771/Scr control cells (Fig. 2b). Although all control isografts growing in syngeneic mice after 21 days expressed Plac1, there was little residual staining in mammary tissue from EO771/shPlac1-engrafted mice (Fig. 2c). Although Plac1 reduced growth *in vitro*, the lack of sustained growth of EO771/shPlac1 cells in syngeneic mice suggested that interactions with the tumor microenvironment may have contributed to this effect.

### Cxcr2 antagonist inhibits tumor growth

**Figure 3:**
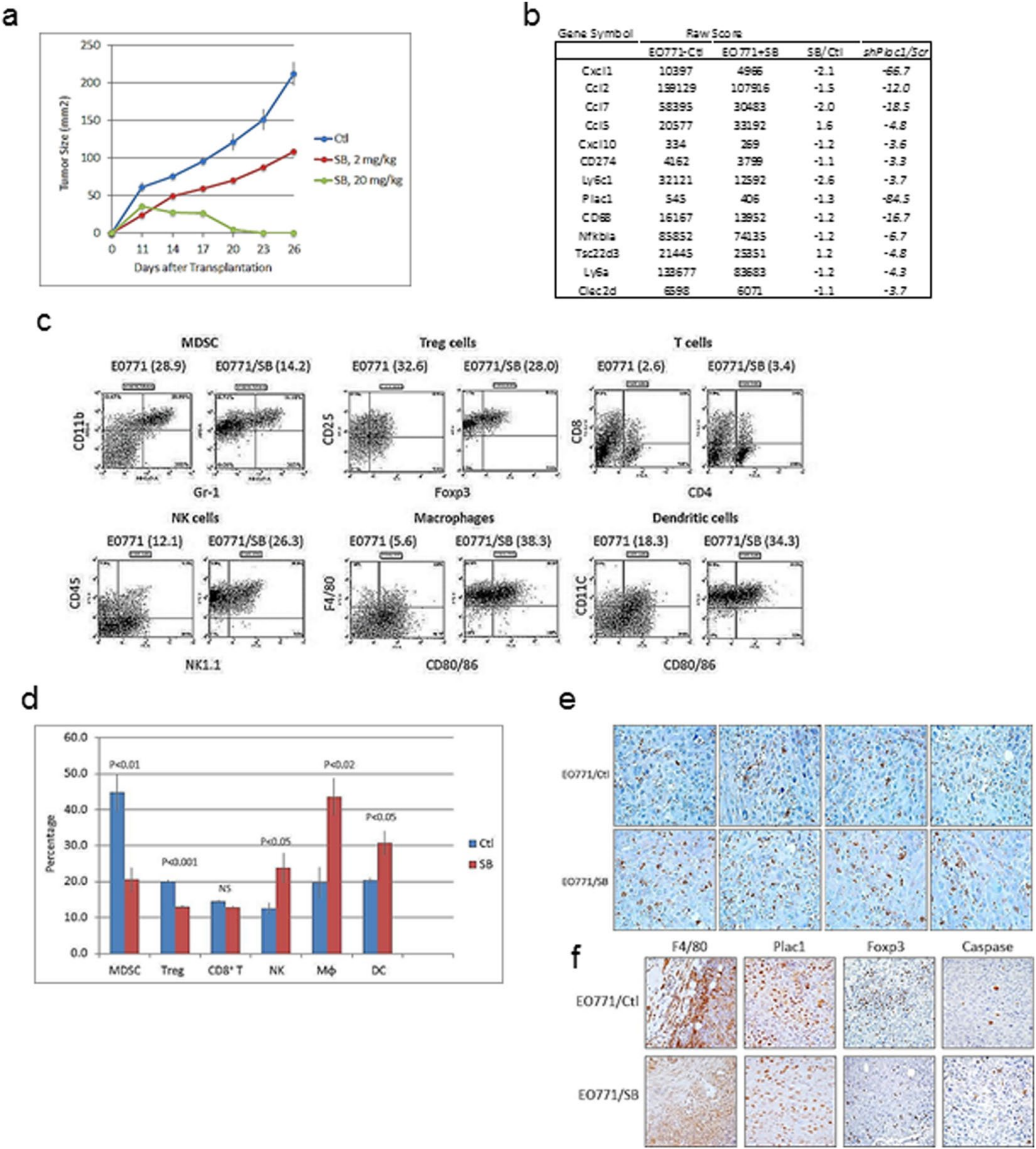
Growth of EO771 cells in syngeneic mice following treatment with a Cxcr_2_ antagonist. (**a**) Syngeneic 57BL/6 mice were inoculated in the mammary gland with 1 × 10^6^ at five weeks of age, and injected i.p. daily with vehicle (blue) or 2 mg/kg (red) or 20 mg/kg (green) SB225002 beginning 11 days after cell inoculation. SB225002 completely suppressed tumor growth after 14 days. Differences between vehicle- and 2 mg/kg SB225002-treated mice were not significantly different (P = 0.145); differences between vehicle- and 20 mg/kg SB225002-treated mice were significantly different (P = 0.005) by the unpaired two-tailed Student’s t test. Shown is the mean ± SD, N = 5 per group. (**b**) Immune gene expression in tumors 17 days after treatment with 20 mg/kg SB225002. Shown is the relative expression in control and SB225002-treated mice in comparison to their changes in EO771/shPlac1 cells (Table 1). (**c**) FACS analysis of immune cell tumor infiltrates in isografts after treatment with vehicle or SB225002 as in (**b**). SB225002 treatment reduced the percentage of immune cell tumor infiltrates of CD11b^+^/Gr-1^+^ myeloid-derived suppressor cells (*MDSC*) and Foxp3^+^/CD25^+^ T cells (*Treg*), and increased the percentages of CD8^+^/CD4^+^ T cells (*T)*, CD3^+^/NK1.1^+^ NK cells (*NK*) and F4/80^+^/CD80/86^+^ macrophages (*Mϕ*) and CD11c^+^/CD80/86^+^ dendritic cells (*DC*). Numbers in parentheses ( ) represent the percentages of each cell population. (**d**) Bar graph represents the mean±SD of the percent distribution of immune cell tumor infiltrates as in (**c**); P values were determined by the unpaired two-tailed Student’s t test, N = 4 per group. (**e**) CD8^+^ T cell infiltration determined by IHC in tumor isografts from vehicle-treated (*EO771/Ctl*) and SB225002-treated (*EO771/SB*) mice. Infiltration of CD8^+^ T cells increased after treatment with 20 mg/kg SB225002. Magnification 600X. (**f**) Macrophage (*F4/80*) and Treg cell (*Foxp3*) infiltration, Plac1 expression and apoptosis by cleaved caspase-3 expression (*Caspase*) in tumor isografts from vehicle-treated (*EO771/Ctl*) and SB225002-treated (*EO771/SB*) mice. Infiltration of macrophages and Treg cells were reduced and apoptosis was increased after treatment with 20 mg/kg SB225002. Magnification 400X

Since Cxcl1 exhibited the greatest change in expression among all chemokine genes after Plac1 downregulation (Table 1), mice were treated with a Cxcr2 antagonist to determine if it would affect tumor growth to a similar extent. Animals engrafted with EO771 cells were treated three times per week with vehicle or 2 or 20 mg/kg SB225002 beginning 10 days after transplantation (Fig. 3a). Whereas, the lower dose partially inhibited tumor growth, 20 mg/kg SB225002 completely blocked growth after a lag of two weeks. Tumor stasis at 17 days following cell inoculation was associated with reduction in expression of immune and chemokine genes, many of which were downregulated in EO771/shPlac1 cells (Fig. 3b). Cell sorting of tumor immune infiltrates indicated that SB225002 treatment reduced myeloid-derived suppressor cells (*MDSC*) and Treg cells, and increased CD8^+^/CD4^+^ T cells, NK cells, macrophages and dendritic cells (Fig. 3c,d). Especially noteworthy was the greater infiltration of CD8^+^ T cells into the tumor bed following SB25002 treatment (Fig. 3e), which was accompanied by increased macrophage and Treg cell infiltration, reduction of Plac1 and increased apoptosis (Fig. 3f). Since we did not determine tissue levels of SB25002, we determined its cytotoxicity in EO771 cell culture (Supplementary Fig. 2). SB25002 at concentration less than 100 nM were not cytotoxic, but produced cytotoxicity at concentrations exceeding 1000 nM. Since we have not carried out pharmacokinetics, the contribution of SB25002 cytotoxicity to its antitumor effect cannot be ascertained.

### Cxcl1 reduction inhibits tumor growth and immune cell-related transcription

**Figure 4:**
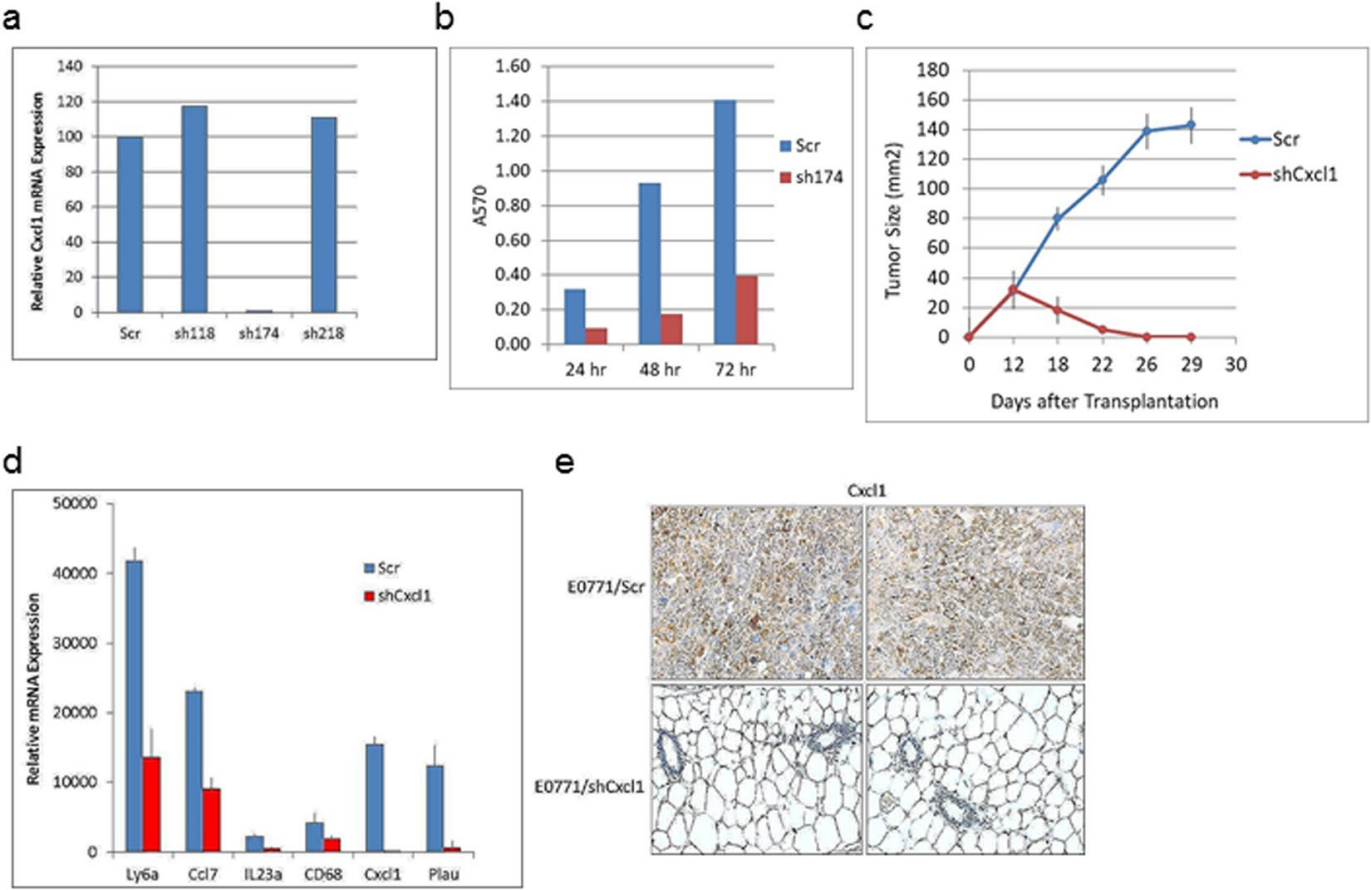
Lentivirus-mediated reduction of Cxcl1 in EO771 cells. (**a**) EO771 cells were transduced with lentiviruses expressing scrambled RNA (Scr) or three Cxcl1 shRNAs designated sh118, sh174, sh218; sh174 inhibited RNA expression >99% (EO771/shCxcl1). (**b**) EO771/Scr and EO771/shCxcl1 cells were grown as monolayers, and the number of viable cells were determined by sulforhodamine B staining. Shown is the mean ± S.D. of triplicate analysis from three samples, which were significantly different (*P* < 0.001) by the two-tailed Student’s t test. (**c**) Growth of EO771/Scr and EO771/shCxcl1 cells in syngeneic mice. Mice at five weeks of age were inoculated in the mammary gland with 1x10^6^ cells, and tumor size was measured by calipers in two dimensions. Differences in tumor growth between EO771/Scr and EO771/shCxcl1 cells were significantly different (P = 0.006) by the unpaired two-tailed Student’s t test; N = 5. (**d**) qRT-PCR analysis of genes downregulated in EO771/shCxcl1 cells. Shown is the mean ± SD of triplicate analysis of 3 samples. Significant differences between EO771/Scr and EO771/shCxcl1 cells were obtained for Plau (*P* < 0.02), C3 (*P* < 0.01), Ly6a (*P* < 0.01), Ccl7 (*P* < 0.001) and Il23a (*P* < 0.01) by the two-sided Student’s t test; differences for CD68 were not significantly different (*P* > 0.05).

To evaluate the role of Cxcl1/Cxcr2 signaling in tumor growth, EO771 cells were transduced with scrambled shRNA (Scr) or four Cxcl1 shRNAs (Fig. 4a). EO771 cells expressing sh174 (*shCxcl1*) exhibited >95% reduction of Cxcl1 RNA expression. After 48 hr in monolayer culture, EO771/shCxcl1 cells grew at approximately 30% of the rate of control cells (*Scr*) (Fig. 4b). Comparison of the gene expression profile of EO771/shCxcl1 cells with EO771/Scr cells revealed a small subset of genes with ≥3-fold changes, including Ly6a, IL23a, C3, Cxcl1 and CD68 (Table 2, Supplementary Table 3). Transplantation of EO771/shCxcl1 cells into syngeneic mice resulted in impaired tumor growth in comparison to control cells (Fig. 4c), a result that was similar to EO771/shPlac1 cells (Fig. 2a). Changes in immune-related gene expression (Table 2) were confirmed by qRT-PCR, with the exception of CD68, which did not change significantly (Fig. 4d). Measurement of Cxcl1 in tumors or mammary tissue by IHC after 21days indicated the presence of Cxcl1 in EO771/Scr tumors, but not in EO771/shCxcl1 engrafted mammary tissue (Fig. 4e). Comparison of gene expression in EO771/shPlac1 vs. EO771/shCxcl1 cells indicated reduced expression in five genes in common, viz. CD68, Cxcl1, Ly6a, Plau and Rgs16 (Table 3), although CD68 was not changed significantly in EO771/shCxcl1 cells as measured by qRT-PCR (Fig. 4d).

**Table 2:** Expression of immune-related genes in E0771/shCxcl1 cells. Shown are ≥3-fold changes in gene expression with a raw score ≥300 in EO771/shCxcl1 or E0771/Scr cells.

Gene	log 2		Raw Score		shCxcl1/Scr
	Scr	shCxcl1	Scr	shCxcl1	
Ly6a	12.93	11.29	7787	2512	−3.1
Il23a	8.84	7.06	459	133	−3.5
C3	8.48	6.55	358	94	−3.8
Cxcl1	11.06	9.07	2137	536	−4.0
CD68	8.62	6.54	394	93	−4.2

**Table 3:** Gene expression common to EO771/shPlac1 and EO771/shCxcl1 cells. Shown is the ratio between EO771/shPlac1 or EO771/shCxcl1 cells to EO771/Scr control cells for genes with ≥3.0-fold changes in expression and a raw score ≥300.

Gene	shPlac1/Scr	shCxcl1/Scr
CD68	−4.2	−4.2
Cxcl1	−67	−4.0
Ly6a	−4.3	−3.1
Plau	−7.1	−7.3
Rgs16	−7.7	−3.2

### Cxcl1 partially rescues Plac1 reduction in EO771 cells

**Figure 5:**
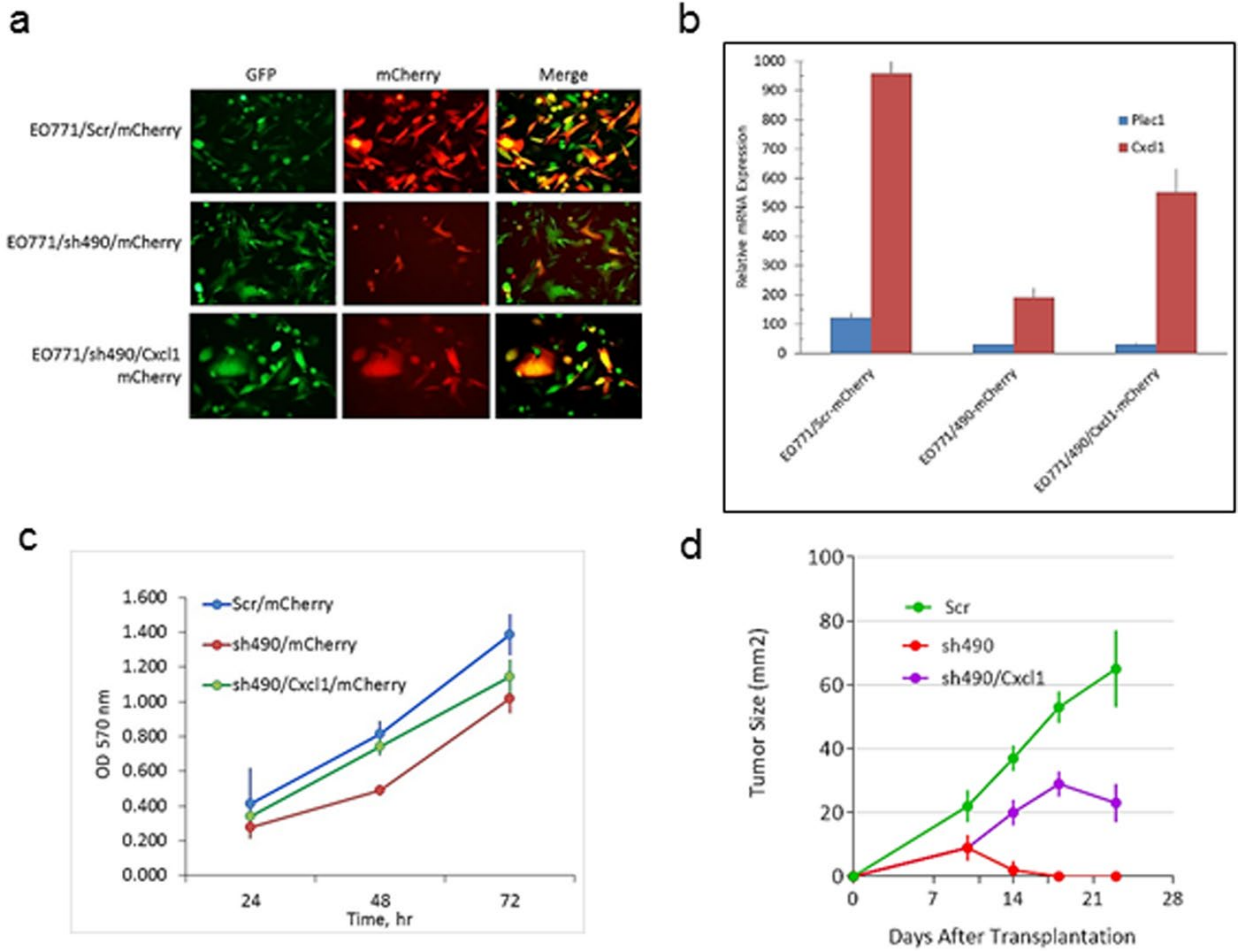
Cxcl1 rescue of EO771/sh490 cells. (**a**) EO771/Scr and EO771/sh490 cells expressing eGFP were transduced with a lentivirus expressing Cxcl1 and mCherry, and selected for 35 days in 3.5 mg/ml G418. The merged photo shows cells co-expressing eGFP and mCherry (yellow). Magnification 200X. (**b**) qRT-PCR for Plac1 and Cxcl1 in EO771/Scr, EO771/sh490 and EO771/sh490/Cxcl1 cells. Shown is the mean ± S.D. of triplicate determinations.(**c**) EO771/sh490/Cxcl1 cells were grown in 96-well plates at an initial density of 5,000 cells per well in media supplemented with 3.5 mg/ml G418. Cell density was determined by sulforhodamine B staining. Shown is the mean ± SD of triplicate determinations. (**d**) Syngeneic C57BL/6 mice were inoculated in the mammary gland with 1 × 10^6^ at five weeks of age. There was a significant difference in the growth EO771/sh490 cells (P = 0.021) and EO771/sh490/Cxcl1 cells (P = 0.034) vs. EO771/Scr cells by the unpaired two-tailed Student’s t test. Shown is the mean ± SD, N=6 per group.

To determine the contribution of Cxcl1 to the effects of Plac1 downregulation on tumor growth, EOI771/sh490 cells were transduced with a retrovirus expressing Cxcl1-mCherry and EO771/Scr and EO771/sh490 were transduced with mCherry alone (Fig. 5). After selection in G418, a significant percentage of cells co- expressed GFP and mCherry (Fig. 5a) and Cxcl1 mRNA (Fig. 5b). The growth of EO771/sh490 cells *in vitro* was slower rate than control cells as shown in Fig. 1c, but cells expressing Cxcl1 largely rescued this effect (Fig. 5c). Isografts of these cell lines in syngeneic mice confirmed the poor growth of EO771/sh490 cells, and further showed that Cxcl1 could partially rescue their poor tumorigenicity (Fig. 5d).

As an added proof of the function of Plac1 in tumorigenesis, MC cells, a cell line with low Plac1 expression (Fig. 1a), were transfected with Plac1 (Supplementary Fig. 3, Supplementary Table 5). MC/Plac1 cells grew at a higher rate than control cells and exhibited less apoptosis, and upregulated a gene expression profile that included several of the chemokines as well as CD274 that were downregulated in EO771/shPlac1 cells.

## Discussion

The present study establishes the first link between the trophoblast gene, Plac1, and adaptive immunity, through its ability to modulate chemokine expression and other immune cell regulators. In retrospect, this is not too surprising since the placenta may be regarded as a foreign allograft protected from host vs. graft rejection^23^ in part by the presence of Treg cells in the uterine decidua^24^. However, the link between Plac1, chemokine signaling and immune tolerance in tumors is a novel and relevant finding since the latter processes are hallmarks of most, if not all, solid tumors^25,26^. The relevance of Plac1 to mammary tumorigenesis was first noted in MMTV-PPARd mice, where Plac1 was markedly upregulated at the onset and throughout tumor development^14^. This finding implicated nuclear receptor signaling in the transcriptional regulation of Plac1, as noted previously for its activation at alternate promoter regions in the *Plac1* locus by LXR and RXRA^27^. However, from a mechanistic perspective, the downstream intracellular components interacting with Plac1have not been determined. Plac1 is predominantly extracellular with an N-terminal signal peptide, a small transmembrane domain and an extracellular ZP3 domain^28^, which promotes protein-protein interactions. By analogy, cytokine receptors lacking an intracellular signaling domain partner with co-receptors, adapter molecules and cytosolic protein tyrosine kinases to effect signaling^29^, and such a mechanism may also pertain to Plac1.

In the present study, the panel of immune-related genes down-regulated by Plac1 ‘knockdown’ (Table 1) suggest that one of its functions is to modulate chemokine effector pathways associated with immune evasion, such as antigen presentation, angiogenesis and myeloid cell, T cell and fibroblast activation (see scheme in Supplementary Fig. 4). One dominant downstream effector pathway was the Cxcl1/Cxcr2 axis, as shown by inhibition of tumor growth by the Cxcr2 antagonist SB25002 (Fig. 3a), and the inability of EO771/shCxcl1 cells to sustain tumor proliferation in syngeneic mice (Fig. 4c). Inhibition of Cxcr2 by SB25002 was associated with reduction of Treg cells and MDSC, and an increasing the percentage of CD8^+^ T cells, macrophages, NK cells and dendritic cells, which was consistent with previous studies demonstrating the ability of SB225002 to suppress MDSC infiltration in breast tumor xenografts^30^ and prostate tumors^31^, as well as metastasis via S100A8/A9^30^. Thus, our data suggest that one role of Plac1 may be to maintain the production of inflammatory and immunoregulatory chemokines to effect changes in the stromal microenvironment conducive to immune tolerance and poor outcome^30,32,33^, which would explain the poor tumorigenicity of EO771/shPlac1 cells in syngeneic mice, but not in SCID mice. Although, co-expression of Plac1 and Cxcl1 in breast cancer tissue has not been reported, Cxcl1 expression in breast cancer biopsies was found to be elevated in metastases, and inversely related to ERα expression and relapse-free survival^34^.

Despite focusing on the immunological aspects of Plac1 function, it was apparent that it affected several signaling pathways in EO771 cells (Supplementary Table 2). Changes in gene expression common to both EO771/shPlac1 and EO771/shCxcl1 cells, apart from Cxcl1, included reduced expression of Plau, Ly6a, CD68 and Rgs16 (Table 3). Plau is a well-known marker of metastasis^35^, and its role in invasion has been noted in trophoblast migration^36^. Ly6a/Sca-1 is a mouse stem cell and tumor-initiating cell biomarker^37,38^ that down-regulates several tumor suppressor pathways, including PPARγ, TGF-β and PTEN^39,40^. CD68 is a scavenger receptor involved in phagocytosis, particularly in M2 polarized macrophages^41^, and has been implicated in immunotolerance by tumor-associated macrophages^42^. Rgs16 is a G-protein-coupled receptor associated with vascular smooth muscle cell proliferation and angiogenesis, which play prominent roles in oncogenesis^43^. Thus, the functions of Plac1 in placental development^44^ appear to phenocopy its functions in tumorigenesis, which supports the onco-placental nature of cancer^3^. From a therapeutic perspective, our data not only suggest that Plac1 may be a potential drug target, but that chemokine receptor antagonists developed for chronic inflammatory disorders, including COPD and psoriasis^45,46^, may be useful adjuvants when used in combination with other therapies to enhance the efficacy of cancer treatment^47,48^.

## Methods

### Cell lines

EO771 cells were originally isolated from a spontaneous mammary carcinoma in C57BL/6 mice^49^, and were provided by Dr. Louis M. Weiner, Georgetown University. EO771 cells tested negative against the IMPACT II panel of infectious agents (IDEXX). Plac1 and Cxcl1 expression were reduced with the piLenti-sRNA-GFP lentiviral vector targeting the sequence 5′-CCACCTTATGTCTACAATCAAAAGAGCAT-3′ of Plac11 mRNA (cat #i034429, ABM, Vancouver, Canada) or the sequence 5′-CTGCACCCAAACCGAAGTCATAGCCACAC-3′ of Cxcl1 mRNA (cat # i042697, ABM); a scrambled sequence was used as a control. Lentivirus expression of Cxcl1 utilized Lenti ORF clone Cxcl1 (MR220966L1) from Origene and lenti vector CMV-mCxcl1-IRES-mCherry (VB170524-1062) from VectorBuilder or the vector lacking Cxcl1 as a control. HEK293T cells were co-transfected at 50% confluence with the lentiviral shRNA plasmid, psPAX2 packaging plasmid and the VSV-G/pMD2 envelope plasmid at a ratio of 2:1:0.1 using Fugene 6 (Promega). After 18 hr, medium was replaced with fresh growth medium, and after 24–48 hr, the virus-containing supernatant was collected, filtered through a 0.45 um filter, mixed with fresh cell culture medium at a ratio of 7:1, and added to EO771 cells with 8 µg/ml polybrene. A lentivirus expressing a scrambled non-silencing control shRNA (shRNAmir, ABM) served as a negative control. Cells were selected for stable integration of the virus by incubation with 7.5μg/ml puromycin (Sigma-Aldrich Corp.) for 10 days. The efficiency of integration was monitored by GFP expression by the lentivirus. Cell lines 34T, 105T, 437T, MC and NeuT were described previously^50,51^.

### Animals

EO771 cells at an inoculum of 1 × 10^6^ cells/0.1 ml were injected into the no. 4 mammary gland of C57BL/6 or SCID mice (Taconic), and tumor growth was monitored daily. Cxcr2 antagonist SB225002 (Sigma-Aldrich) was dissolved in a diluent containing 8% DMSO, 10% PEG-400 and 1.75% Tween-20 in water at a concentration of 0.4 or 4.0 mg/ml, and administered i.p. Monday through Friday at a dose of 2 or 20 mg/kg, respectively^52,53^. Other mouse mammary tumor cells lines tested for Plac1 expression were 34T, 105T, 437T and MC^40,51^. Animal studies were conducted under protocols approved by the Georgetown University Animal Care and Use Committee (protocol 2016-1143) in accordance with NIH guidelines for the ethical treatment of animals.

### Cell growth and cytotoxicity assays

EO771 or MC cells were grown in 96-well plates at an initial density of 5,000 cells per well. The cytotoxicity of SB225002 was determined in EO771 cells by dissolving the drug in DMSO and diluting in medium to a final DMSO concentration of 0.001%. Cell density was determined after incubation for 24, 48 and 72 hr by sulforhodamine B staining and measuring optical density at 570 nm^54^. Cytotoxicity assay data are shown in Supplementary Fig. 3.

### Histopathology and immunohistochemistry (IHC)

Mammary tumors were excised, and formalin-fixed, paraffin-embedded sections were prepared for H&E staining and IHC by the Tissue and Histopathology Shared Resource, LCCC. Antigen retrieval was carried out by incubation of tissue sections in 10 mM sodium citrate buffer (pH 6.0) for 20 min at a sub-boiling temperature in an electric steamer as previously described^14,51,55^. Endogenous peroxidase activity was quenched with 3% hydrogen peroxide for 10 min, and incubated for 30 min with blocking solution (10% goat serum in Tris-buffered saline), followed by incubation overnight at 4 °C with the appropriate primary antibody diluted in blocking solution. Biotin-conjugated secondary antibodies were diluted in TBS containing 0.1% Tween-20 and incubated for 30 min at room temperature using the ABC Vectastain (Vector Laboratories) detection system and diaminobenzidine (Pierce), and slides were counterstained with Harris-modified hematoxylin (Thermo-Fisher, Inc.), dehydrated and mounted in Permount (Thermo-Fisher, Inc.). Apoptosis was determined with the SignalStain Apoptosis IHC Detection kit for cleaved caspase-3 (Cell Signaling Technology). Antibodies and their dilutions for IHC and FACS are listed in Supplementary Table 1.

### Fluorescence-Activated Cell Sorting (FACS)

Tumor immune infiltrates were obtained by excising tumors, mincing them into small pieces and digestion with collagenase D (Roche) at a ratio of 15 ml collagenase solution per 2 g of tissue for 1 hr at 37 °C with shaking. The cell suspension was filtered through a 70 μm cell strainer (Falcon), washed, erythrocytes lysed and 1 × 10^6^ cells were analyzed by FACS. Cells were stained using the Live/Dead Fixable Dead Cell Stain Kit (Invitrogen) and excluded from analysis, and non-specific binding was blocked with Fc antibody CD16/32 (Biolegend). Cells were first sorted for CD45 (macrophages, MDSC, Treg cells, NK cells and dendritic cells), CD45^+^CD3^+^ (T cells) or CD45^+^CD4^+^ (Treg cells). Cells were further sorted for: macrophages: F4/80^+^/MHCII^+^, MDSC: CD11b^+^/Gr-1^+^, dendritic cells: CD11c^+^/MHCII^+^, T cells: CD4^+^/CD8^+^, NK cells: CD45^+^/NK1.1^+^ and Treg cells: CD25^+^/Foxp3^+^. The fluorescent-conjugated monoclonal antibodies and their dilutions are listed in Supplementary Table 1. Cells were stained for Foxp3 after fixation in 1% paraformaldehyde and permeabilization (Permeabilization Buffer, eBioscience). Flow cytometry data was acquired by the Flow Cytometry & Cell Sorting Shared Resource, LCCC, with a BD LSRFortessa analyzer (BD Biosciences) and FCS Express 4 software (De Novo Software) to determine mean fluorescence intensity.

### Gene microarray analysis

Microarray analysis was carried out as previously described^39,51,55–57^. Briefly, tissue was snap-frozen in liquid nitrogen, pulverized in a mortar and pestle and RNA was extracted using an RNeasy Mini Kit (Qiagen) according to the manufacturer’s protocol. RNA purity was assessed by the integrity of 18S and 28S rRNA using an Agilent microfluidic chip. Array analysis was carried out with cRNA prepared from equal amounts of RNA (1 μg) pooled from three replicates of cells per group. Biotin-labeled cRNA was fragmented at 94°C for 35 min and hybridized overnight to an Affymetrix mouse 430A 2.0 GeneChip^®^, and scanned with an Agilent Gene Array scanner. Grid alignment and raw data generation used the Affymetrix GeneChip^®^ Operating software 1.1. A noise value (*Q*) based on the variance of low-intensity probe cells was used to calculate a minimum threshold for each GeneChip. Samples were averaged and data refined by eliminating genes with signal intensities <300 in both comparison groups, and heat maps were generated from ≥3-fold changes in gene expression normalized to control tissue using unsupervised hierarchical cluster analysis as previously described^58^. Gene expression data for EO771 control, EO881/shPlac1 and EO771/shCxcl1 cells are included in Supplementary Tables 2 and 3, respectively. Gene expression data for mice treated with vehicle or 20 mg/kg SB25001 are included in Supplementary Table 4. Gene interaction analysis utilized Ariadne Pathway Studio version 9.1 (Supplementary Fig. 4). Data sets were deposited in the GEO public database under accession no. GSE78202.

### Quantitative real-time polymerase chain reaction (qRT-PCR)

Total RNA was extracted as described above, and RNA (1 µg) from each of 3 samples per group was reverse transcribed using the Omniscript RT kit (Qiagen) as previously described^39,51,55–57^. PCR was performed in triplicate using an ABI-Prism 7700 (Applied Biosystems, Foster City, CA) with SYBRGreen I detection (Qiagen) according to the manufacturer’s protocol. Amplification using the appropriate primers (Supplementary Table 5) was confirmed by ethidium bromide staining of the PCR products on an agarose gel. The expression of each target gene was normalized to GAPDH and is presented as the ratio of the target gene to GADPH expression calculated using the formula, 2^−ΔCt^, where ΔCt = Ct^Target^ − Ct18s ^39^.

### Statistical analysis

Statistical significance of means ± S.D. were evaluated using the two-tailed Student’s t test at a significance of *P* < 0.05. Differences in tumor growth *in vivo* were determined by the unpaired two-tailed Student’s t test at a significance of *P* < 0.05.

## Electronic supplementary material

Supplementary Information
