# Supplementary material for: Plac1 Is a Key Regulator of the Inflammatory Response and Immune Tolerance In Mammary Tumorigenesis
Source: Sci Rep. 2018 Apr 9;8:5717. doi: 10.1038/s41598-018-24022-w (PMC5890253; doi:10.1038/s41598-018-24022-w)
Supplement: Supplementary file 1 — Supplementary Information [file 41598_2018_24022_MOESM1_ESM.pdf]

# Plac1 Is a Key Regulator of the Inflammatory Response and Immune Tolerance In Mammary Tumorigenesis

Hongyan Yuan, Xiaoyi Wang, Chunmei Shi, Lu Jin, Jianxia Hu, Alston Zhang, James Li, Nairuthya Vijayendra, Venkata Doodala, Spencer Weiss, Yong Tang, Louis M. Weiner and Robert I. Glazer.

## Supplementary Data

Supplementary Figure 1. EO771/sh490/sh187 Plac1 tumor growth

Supplementary Figure 2. SB225002 cytotoxicity assay

Supplementary Figure 3. MC/Plac1 cells

Supplementary Figure 4. Immune signaling pathways

Supplementary Table 1. Antibodies for IHC and FACS

Supplementary Table 2. Gene expression in EO771/shPlac1 cells

Supplementary Table 3. Gene expression in EO771/shCxcl1 cells

Supplementary Table 4. Gene expression in EO771 tumors+SB25002 treatment

Supplementary Table 5. Gene expression for MC/Plac1 cells

Supplementary Table 6. List of primers for qRT-PCR analysis.

a b

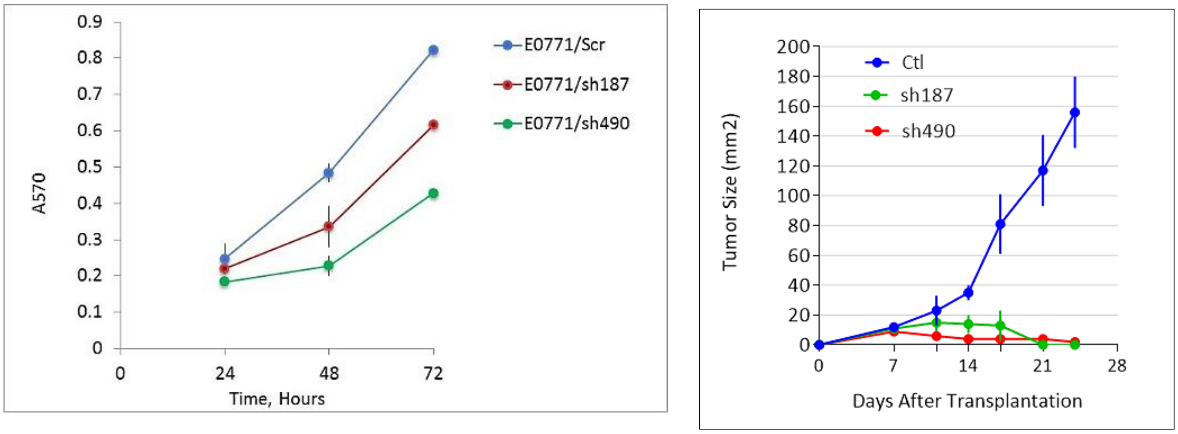


**Supplementary Figure 1. Growth of EO771/Scr, EO771/sh187 and EO771/sh490 cells, and the effect of Cxcl1 co-expression in EO771/sh490 cells**. (**a**) EO771/Scr, EO771/sh187 and EO771/sh490 cells were grown as monolayers and cell density determined by sulforhodamine B staining and measuring the absorbance at 570 nm. Shown is the mean±S.D. of triplicate determinations. The growth of EO771/sh490 cells differed significantly ($P<0.001$) from EO771/Scr cells at 24-72 hr using the two-sided Student's t test. (**b**) C57BL/6 mice at five weeks of age, were inoculated in the mammary gland with $1x10^6$ cells, and tumor size determined by caliper measurement in two dimensions. The growth of EO771/sh187 or EO771/sh490 cells differed significantly from EO771/Scr ($P<0.01$) by the unpaired Student's t test. Shown is the mean±SD, N=6 per group.

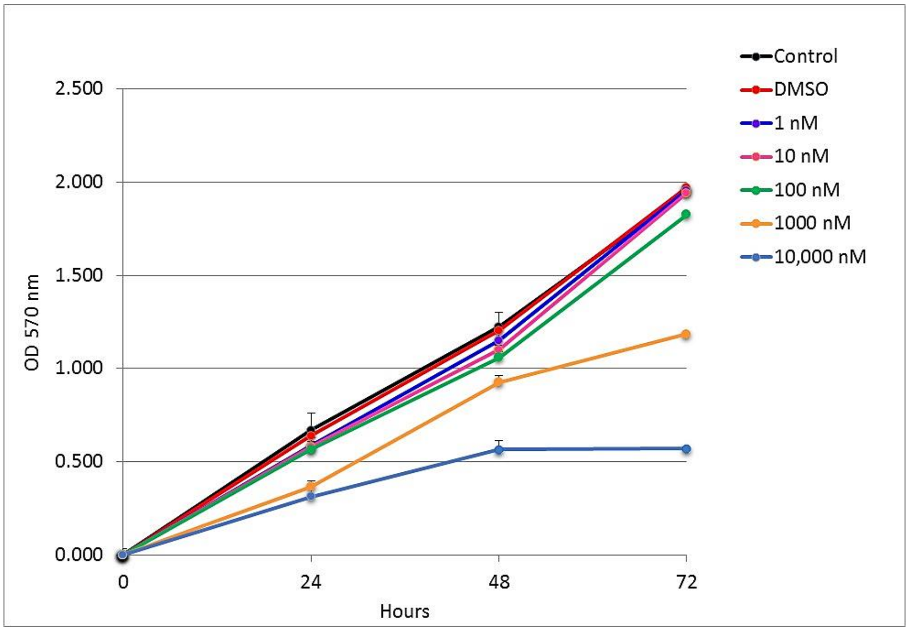


**Supplementary Figure 2. In vitro cytotoxicity of SB225002 to EO771 cells.** EO771 cells were grown in 96-well plates at an initial density of 5,000 cells per well, and treated with 0.001% DMSO or SB225002. Cell density was determined after 24, 48 and 72 hr by sulforhodamine B staining and measuring absorbance at 570 nm. Indicated is the final concentration (nM) of SB225002. Shown is the mean ± SD of triplicate determinations.

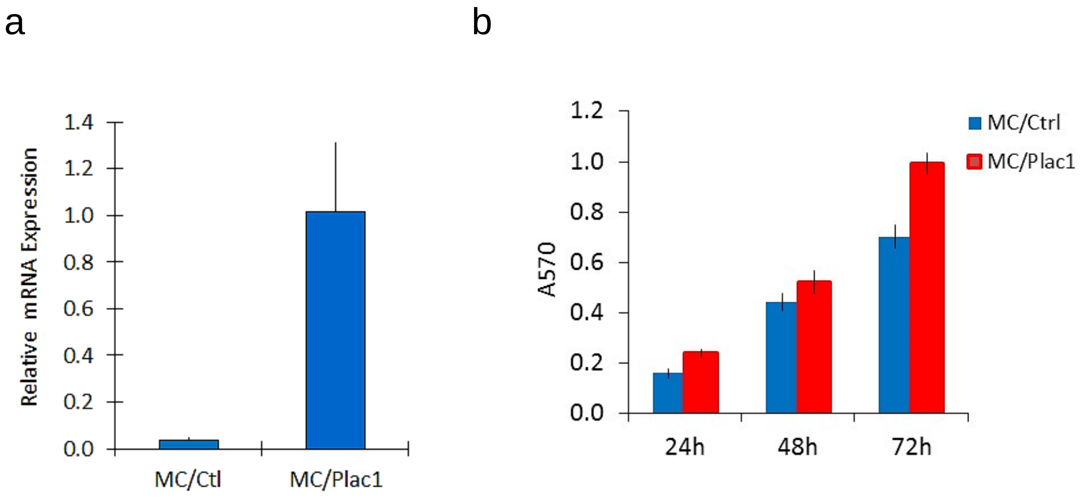


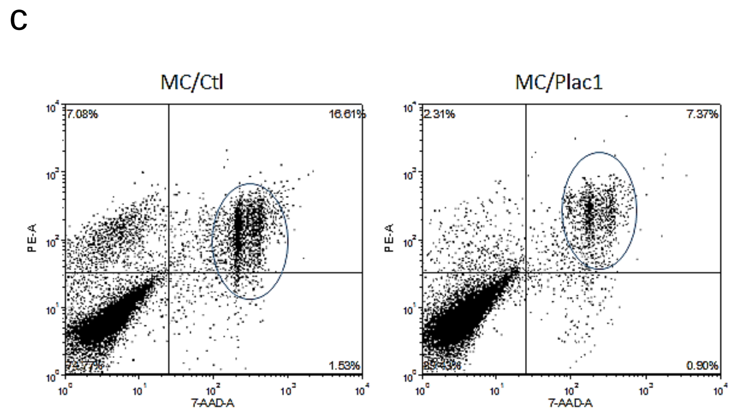


d

| | FC | Raw Score | |
|---|---|---|---|
| **Gene** | **Plac1/Ctl** | **MC/Ctl** | **MC/Plac1** |
| Cxcl11 | 37.0 | 38 | 1409 |
| Cxcl10 | 13.1 | 154 | 2020 |
| Ccl5 | 7.1 | 1872 | 13216 |
| Ccl2 | 4.7 | 184 | 861 |
| Il33 | 5.4 | 73 | 399 |
| Cd274 | 3.0 | 163 | 491 |

**Supplementary Figure 3. Overexpression of Plac1 in MC mammary tumor cells.** (**a**) qRT-PCR analysis of MC/Plac1 cells transfected with a vector expressing murine Plac1 and selected in G-418. MC/Plac1 cells expressed significantly greater levels of Plac1 than control MC/Ctl cells by the two-sided Student's t test (P<0.001, N=3). (**b**) MC/Plac1 cells were grown in a 96-well plate at an initial density of 5,000 cells per well for 24-72 hr, and cell density determined by sulforhodamine B staining and measuring absorbance at 570 nm. Shown is the mean ± SD of triplicate determinations. (**c**) Analysis of apoptosis in MC/Plac1 cells by fluorescence of 7-aminoactinomycin D as a measure of cell viability. The scatter determined by FACS is *circled* and is a measure of late apoptosis, which decreased from 16.6% in MC/Ctl cells to 7.3% in MC/Plac1 cells. (**d**) Table of chemokine and immune-related gene expression in MC/Ctl and MC/Plac1 cells as determined by Affymetrix gene expression profiling (see Supplementary Table 5 for full list).

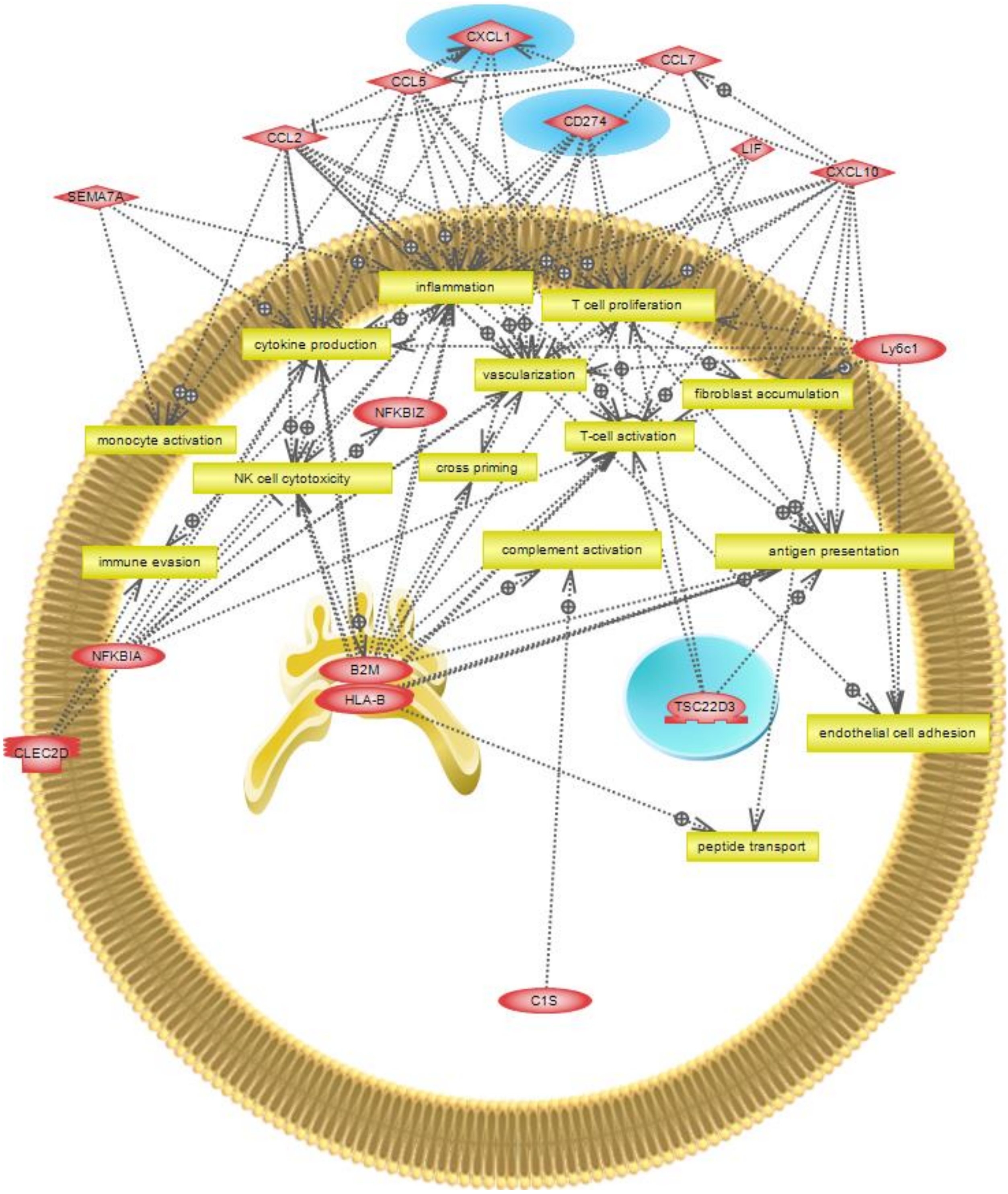


**Supplementary Figure 4. Schematic of immune signaling by Plac1 in EO771 cells.** Based on data in Table 1, EO771 cells overexpress several chemokines and immune factors, which regulate inflammation, cytokine production, fibroblast activation, antigen presentation, and monocyte and T cell activation. Plac1 maintained high CXCL1 expression, a chemokine that activates CXCR2 on MDSC, whereas, CD274/ PD-L1 expressed by MDSC and tumor cells activates Treg cells; both pathways contribute to immune tolerance. Inhibition of CXCR2 with an antagonist and treatment with a PD-L1 mAb counteracts in part activation of a tolerogenic tumor microenvironment.

**Supplementary Table 1. Antibodies for IHC and FACS.**

| | Catalog # | Source | Dilution IHC | Dilution FACS |
|---|---|---|---|---|
| anti-Plac1 | sc-365919 | Santa Cruz | 400 | |
| anti-PD-L1 | 17952-I-AP | Proteintech | 200 | |
| anti-Cxcl1 | sc-1374 | Santa Cruz | 120 | |
| anti-CD8a | 14-0808-82 | eBioscience | 40 | |
| anti-SMA | sc130617 | Santa Cruz | 100 | |
| anti-Ki67 | CRM325 | Biocare | 50 | |
| anti-CD31 | ab56299 | abcam | 100 | |
| anti-Foxp3 | 14-5773-82 | eBioscience | 50 | |
| CD16/32 | 101301 | Biolegend | | 100 |
| FITC anti-mouse CD4 | 100509 | Biolegend | | 200 |
| PE/Cy7 anti-mouse CD8a | 100721 | Biolegend | | 40 |
| APC anti-mouse CD3 epsilon | 100311 | Biolegend | | 100 |
| APC anti-mouse CD25 | 102011 | Biolegend | | 400 |
| PE anti-mouse CD80 | 104707 | Biolegend | | 400 |
| PE anti-mouse CD86 | 105007 | Biolegend | | 125 |
| APC anti-mouse F4/80 | 123115 | Biolegend | | 80 |
| APC/Cy7 anti-mouse CD45 | 103115 | Biolegend | | 80 |
| PE anti-mouse NK1.1 | 108707 | Biolegend | | 80 |
| APC anti-mouse CD11c | 117309 | Biolegend | | 80 |
| APC anti-mouse/human CD11b | 101211 | Biolegend | | 80 |
| PE anti-mouse Foxp3 | 12-4771-80 | eBioscience | | 80 |
| PE/Cy7 anti-mouse Ly6g/Ly6c (Gr-1) | 108415 | Biolegend | | 200 |

**Supplementary Table 2. Gene expression in EO771/shPlac1 cells.** Shown are genes with ≥3-fold change in expression and a raw score ≥300 in EO771/shPlac1 or EO771/Scr cells.

| | log2 | | Raw Score | | FC | |
|---|---|---|---|---|---|---|
| **Function** | **Scr** | **shPlac1** | **Scr** | **shPlac1** | **shPlac1/Scr** | **Gene Name** |
| **Adhesion/ECM** | | | | | | |
| Pkp2 | 6.63 | 8.27 | 99 | 308 | 3.1 | plakophilin 2 |
| P4ha2 | 11.33 | 9.66 | 2567 | 811 | -3.1 | prolyl 4-hydroxylase, alpha polypeptide II |
| Sdc1 | 11.58 | 9.88 | 3064 | 939 | -3.2 | syndecan 1 |
| Col18a1 | 10.35 | 8.42 | 1301 | 343 | -3.7 | collagen, type XVIII, alpha 1 |
| **Apoptosis** | | | | | | |
| Birc5 | 8.74 | 10.53 | 427 | 1478 | 3.5 | baculoviral IAP repeat containing 5; Survivin |
| Ebag9 | 6.75 | 8.43 | 107 | 345 | 3.2 | estrogen receptor binding site associated, antigen, 9 |
| CD82 | 10.83 | 8.96 | 1823 | 498 | -3.7 | Kangai 1 (suppression of tumorigenicity 6, prostate; |
| Trp53inp1 | 8.30 | 6.34 | 314 | 81 | -3.8 | tumor protein p53 inducible nuclear protein 1 |
| **Differentiation** | | | | | | |
| Pter | 6.83 | 8.62 | 114 | 394 | 3.5 | phosphotriesterase related |
| Krt8 | 10.83 | 12.42 | 1817 | 5489 | 3.0 | keratin 8 |
| Gdf15 | 9.71 | 8.12 | 840 | 279 | -3.3 | growth differentiation factor 15 |
| **DNA Repair** | | | | | | |
| Fignl1 | 7.05 | 9.56 | 132 | 757 | 5.7 | fidgetin-like 1 |
| Rad51 | 6.51 | 8.75 | 91 | 431 | 4.7 | RAD51 recombinase |
| Fen1 | 6.89 | 9.08 | 119 | 540 | 4.6 | flap structure-specific endonuclease 1 |
| Rad51ap1 | 6.37 | 8.51 | 83 | 365 | 4.5 | RAD51 associated protein 1 |
| Ercc6l | 6.71 | 8.83 | 105 | 456 | 4.4 | excision repair cross-complementation group 6-like |
| Rad54l | 6.24 | 8.25 | 75 | 305 | 4.1 | RAD54-like (S. cerevisiae) |
| Ddit3 | 10.19 | 8.16 | 1166 | 286 | -4.0 | DNA-damage-inducible transcript 3 |
| Gadd45b | 10.55 | 7.31 | 1499 | 159 | -12.5 | growth arrest and DNA-damage-inducible, beta |
| **DNA Synthesis** | | | | | | |
| Mki67 | 7.10 | 9.99 | 137 | 1020 | 7.4 | marker of proliferation Ki-67 |
| Tyms | 5.99 | 8.56 | 64 | 378 | 5.9 | thymidylate synthetase |
| Mcm5 | 6.42 | 8.87 | 86 | 467 | 5.4 | minichromosome maintenance complex component 5 |
| Cdc6 | 6.00 | 8.36 | 64 | 328 | 5.1 | cell division cycle 6 |
| Rrm2 | 9.22 | 11.28 | 596 | 2487 | 4.4 | ribonucleotide reductase M2 |
| Rrm1 | 8.32 | 10.34 | 320 | 1296 | 4.1 | ribonucleotide reductase M1 |
| Pola1 | 7.15 | 9.10 | 142 | 549 | 3.9 | polymerase (DNA directed), alpha 1, catalytic subunit |
| Mcm3 | 8.10 | 9.88 | 274 | 942 | 3.5 | minichromosome maintenance complex component 3 |
| Mcm4 | 8.11 | 9.90 | 276 | 955 | 3.5 | minichromosome maintenance complex component 4 |
| Prim1 | 7.73 | 9.47 | 212 | 709 | 3.4 | primase, DNA, polypeptide 1 (49kDa) |
| Nasp | 8.10 | 9.73 | 275 | 850 | 3.1 | nuclear autoantigenic sperm protein (histone-binding) |
| Mcm7 | 8.85 | 10.45 | 462 | 1399 | 3.0 | minichromosome maintenance complex component 7 |
| Smarcb1 | 7.83 | 9.43 | 228 | 690 | 3.0 | SWI/SNF related, matrix associated, actin dependent regulator of chromatin, subfamily b, member 1 |
| **Immune/Inflammation** | | | | | | |
| Tapbp | 11.71 | 10.11 | 3356 | 1102 | -3.0 | TAP binding protein (tapasin) |
| CD274 | 8.24 | 6.50 | 302 | 91 | -3.3 | programmed cell death 1 ligand 1 |
| Cxcl10 | 8.84 | 7.02 | 459 | 130 | -3.6 | chemokine (C-X-C motif) ligand 10 |
| Sema7a | 8.74 | 6.93 | 426 | 122 | -3.6 | semaphorin 7A, GPI membrane anchor (John Milton Hagen blood group) |
| Ifi44 | 9.35 | 7.50 | 652 | 181 | -3.6 | interferon-induced protein 44 |
| Ly6c1 | 10.23 | 8.36 | 1198 | 329 | -3.7 | lymphocyte antigen 6 complex, locus C |
| Clec2d | 8.51 | 6.62 | 366 | 99 | -3.7 | C-type lectin domain family 2, member D |
| Nfkbiz | 9.22 | 7.17 | 596 | 144 | -4.4 | nuclear factor of kappa light polypeptide gene enhancer in B-cells inhibitor, zeta |
| Ly6a | 12.27 | 10.18 | 4937 | 1160 | -4.3 | lymphocyte antigen 6 complex, locus A, stem cell antigen-1 |
| H2-K1 | 11.02 | 8.90 | 2069 | 478 | -4.5 | major histocompatibility complex, class I, K |
| Tsc22d3 | 11.15 | 8.90 | 2265 | 478 | -4.8 | TSC22 domain family, member 3 |
| Ccl5 | 11.90 | 9.61 | 3812 | 784 | -4.8 | chemokine (C-C motif) ligand 5 |
| H2-D1 | 11.20 | 8.88 | 2353 | 471 | -5.0 | major histocompatibility complex, class I, D |
| B2m | 8.58 | 6.16 | 383 | 72 | -5.3 | beta-2-microglobulin |
| Nfkbia | 12.08 | 9.02 | 4330 | 517 | -7.1 | nuclear factor of kappa light polypeptide gene enhancer in B-cells inhibitor, alpha |
| C1s | 9.84 | 7.00 | 918 | 128 | -7.1 | complement component 1, s subcomponent |
| Lif | 9.37 | 6.32 | 664 | 80 | -8.3 | leukemia inhibitory factor |
| Mndal | 10.63 | 7.53 | 1585 | 185 | -8.3 | myeloid cell nuclear differentiation antigen |
| Ccl2 | 11.83 | 8.25 | 3640 | 305 | -11.9 | chemokine (C-C motif) ligand 2 |
| CD68 | 9.86 | 5.78 | 929 | 55 | -16.9 | scavenger receptor class D, member 1 |
| Ccl7 | 11.48 | 7.25 | 2854 | 153 | -18.5 | chemokine (C-C motif) ligand 7 |
| Cxcl1 | 12.78 | 6.73 | 7054 | 106 | -66.7 | chemokine (C-X-C motif) ligand 1 (melanoma growth stimulating activity, alpha) |
| **Invasion/Motility** | | | | | | |
| Diap3 | 5.59 | 8.59 | 48 | 386 | 9.1 | diaphanous-related formin 3 |
| Hmmr | 7.51 | 9.33 | 182 | 644 | 3.5 | hyaluronan-mediated motility receptor (RHAMM) |
| Racgap1 | 8.47 | 10.21 | 355 | 1184 | 3.3 | Rac GTPase activating protein 1 |
| Serpine1 | 10.98 | 9.35 | 2021 | 651 | -3.1 | serpin peptidase inhibitor, clade E (nexin, plasminogen activator inhibitor type 1), member 1 |
| Ntn1 | 9.52 | 7.84 | 736 | 229 | -3.2 | netrin 1 |
| Serpinf1 | 11.60 | 9.75 | 3097 | 860 | -3.4 | serpin peptidase inhibitor, clade F (alpha-2 antiplasmin, pigment epithelium derived factor), member 1 |
| Ctsh | 10.92 | 8.47 | 1931 | 354 | -5.6 | cathepsin H |
| Plau | 9.34 | 6.31 | 646 | 80 | -7.1 | plasminogen activator, urokinase |
| **Metabolism** | | | | | | |
| Pdss1 | 6.40 | 8.33 | 84 | 322 | 3.8 | prenyl (decaprenyl) diphosphate synthase, subunit 1 |
| Mgat2 | 7.70 | 9.33 | 208 | 643 | 3.1 | mannosyl (alpha-1,6-)-glycoprotein beta-1,2-N-acetylglucosaminyltransferase |
| Sephs2 | 7.93 | 9.52 | 244 | 733 | 3.0 | selenophosphate synthetase 2 |
| Npc2 | 13.22 | 11.54 | 9553 | 2987 | -3.2 | Niemann-Pick disease, type C2 |
| Idua | 8.35 | 6.65 | 327 | 100 | -3.2 | iduronidase, alpha-L- |
| ND5 | 11.77 | 9.98 | 3486 | 1008 | -3.4 | mitochondrially encoded NADH dehydrogenase 5 |
| Lipg | 8.32 | 6.54 | 320 | 93 | -3.6 | lipase, endothelial |
| Ip6k1 | 9.86 | 7.72 | 932 | 211 | -4.3 | inositol hexakisphosphate kinase 1 |
| St3gal1 | 8.53 | 6.33 | 370 | 80 | -4.5 | ST3 beta-galactoside alpha-2,3-sialyltransferase 1 |
| Dnpep | 8.79 | 6.46 | 444 | 88 | -5.0 | aspartyl aminopeptidase |
| Fabp4 | 10.76 | 7.33 | 1734 | 161 | -11.4 | fatty acid binding protein 4, adipocyte |

| | | | | | | |
|---|---|---|---|---|---|---|
| **Mitosis** | | | | | | |
| Melk | 6.62 | 8.96 | 98 | 498 | 5.1 | maternal embryonic leucine zipper kinase |
| Nuf2 | 6.74 | 9.05 | 107 | 529 | 5.0 | NUF2, NDC80 kinetochore complex component |
| Cenpf | 7.87 | 10.14 | 233 | 1126 | 4.8 | centromere protein F, 350/400kDa |
| H1f0 | 8.62 | 10.75 | 392 | 1726 | 4.6 | H1 histone family, member 0 |
| Kif22 | 7.69 | 9.83 | 207 | 910 | 4.4 | kinesin family member 22 |
| Ccnb1 | 7.44 | 9.59 | 174 | 771 | 4.2 | cyclin B1 |
| Ndc80 | 6.37 | 8.43 | 83 | 345 | 4.2 | NDC80 kinetochore complex component |
| Kif2c | 7.23 | 9.26 | 150 | 613 | 4.1 | kinesin family member 2C |
| Aspm | 7.42 | 9.45 | 171 | 699 | 4.1 | asp (abnormal spindle) homolog, microcephaly associated (Drosophila) |
| Ccna2 | 9.00 | 10.92 | 512 | 1941 | 3.8 | cyclin A2 |
| Ect2 | 8.08 | 10.01 | 270 | 1030 | 3.8 | epithelial cell transforming 2 |
| Ska1 | 6.72 | 8.63 | 106 | 396 | 3.7 | spindle and kinetochore associated complex subunit 1 |
| Spag5 | 6.65 | 8.42 | 100 | 342 | 3.5 | sperm associated antigen 5 |
| Cdca5 | 7.77 | 9.50 | 219 | 724 | 3.4 | cell division cycle associated 5 |
| Cenpt | 6.81 | 8.55 | 112 | 376 | 3.4 | centromere protein T |
| Nek2 | 7.03 | 8.76 | 130 | 434 | 3.3 | NIMA-related kinase 2 |
| Sgol1 | 7.74 | 9.47 | 214 | 710 | 3.3 | shugoshin-like 1 (S. pombe) |
| Spc25 | 7.44 | 9.18 | 174 | 582 | 3.3 | SPC25, NDC80 kinetochore complex component |
| Tacc3 | 7.88 | 9.59 | 236 | 772 | 3.3 | transforming, acidic coiled-coil containing protein 3 |
| Ncapd2 | 8.00 | 9.71 | 256 | 836 | 3.3 | non-SMC condensin I complex, subunit D2 |
| Ncapg | 7.14 | 8.87 | 141 | 466 | 3.3 | non-SMC condensin I complex, subunit G |
| Ncaph | 7.99 | 9.58 | 255 | 766 | 3.2 | non-SMC condensin I complex, subunit H |
| Aurka | 8.74 | 10.37 | 428 | 1322 | 3.1 | aurora kinase A |
| Tubb2a | 7.48 | 9.10 | 178 | 550 | 3.1 | tubulin, beta 2A class IIa |
| Cdc20 | 10.38 | 11.99 | 1333 | 4063 | 3.1 | cell division cycle 20 |
| Ndrg4 | 10.82 | 9.14 | 1808 | 564 | -3.2 | NDRG family member 4 |
| Plk2 | 9.70 | 7.88 | 830 | 236 | -3.6 | polo-like kinase 2 |
| Pafah1b1 | 8.89 | 7.01 | 474 | 129 | -3.7 | platelet-activating factor acetylhydrolase 1b, regulatory subunit 1 (45kDa) |
| Zwint | 9.11 | 6.72 | 553 | 106 | -5.3 | ZW10 interacting kinetochore protein |
| **Protein Trafficking/Degradation** | | | | | | |
| Diap2 | 5.59 | 8.59 | 48 | 386 | 8.0 | diaphanous-related formin 2 |
| Tubb4b | 10.34 | 12.40 | 1299 | 5409 | 4.2 | tubulin, beta 4B class IVb |
| Dnajc9 | 6.76 | 8.78 | 109 | 440 | 4.1 | DnaJ (Hsp40) homolog, subfamily C, member 9 |
| Uhrf1 | 7.38 | 9.22 | 167 | 598 | 3.6 | ubiquitin-like with PHD and ring finger domains 1 |
| Hspa4 | 8.20 | 9.98 | 294 | 1013 | 3.4 | heat shock 70kDa protein 4 |
| Ide | 7.85 | 9.54 | 231 | 744 | 3.2 | insulin-degrading enzyme |
| Fnbp4 | 8.37 | 6.78 | 330 | 110 | -3.0 | formin binding protein 4 |
| Mapk8ip3 | 8.32 | 6.73 | 319 | 106 | -3.0 | mitogen-activated protein kinase 8 interacting protein 3 |
| Trim30a | 8.45 | 6.81 | 349 | 112 | -3.1 | tripartite motif containing 30 |
| Gramd1a | 10.03 | 8.39 | 1048 | 335 | -3.1 | GRAM domain containing 1A |
| Hspa5 | 13.38 | 11.63 | 10689 | 3170 | -3.3 | heat shock 70kDa protein 5 (glucose-regulated protein, 78kDa) |
| Calr | 11.78 | 10.01 | 3517 | 1031 | -3.4 | calreticulin |
| Rcn3 | 11.45 | 9.61 | 2801 | 780 | -3.6 | reticulocalbin 3, EF-hand calcium binding domain |
| Vcp | 8.26 | 6.36 | 308 | 82 | -3.7 | valosin containing protein |
| Srprb | 9.14 | 7.18 | 564 | 145 | -3.8 | signal recognition particle receptor, B subunit |
| Rab14 | 8.39 | 6.38 | 336 | 83 | -4.0 | RAB14, member RAS oncogene family |
| Tmed3 | 11.31 | 9.24 | 2534 | 604 | -4.2 | transmembrane emp24 protein transport domain containing 3 |
| Arih2 | 9.46 | 7.40 | 707 | 169 | -4.2 | ariadne RBR E3 ubiquitin protein ligase 2 |
| Hspa1b | 8.91 | 6.73 | 481 | 106 | -4.3 | heat shock 70kDa protein 1B |
| Fkbp7 | 9.45 | 7.34 | 700 | 162 | -4.3 | FK506 binding protein 7 |
| Zfand2a | 10.49 | 8.33 | 1438 | 321 | -4.5 | zinc finger, AN1-type domain 2A |
| Arl4d | 10.49 | 8.33 | 1438 | 321 | -5.0 | ADP-ribosylation factor-like 4D |
| Dnajb6 | 9.54 | 7.19 | 746 | 146 | -5.3 | DnaJ (Hsp40) homolog, subfamily B, member 6 |
| Cryab | 10.39 | 7.84 | 1340 | 229 | -5.3 | crystallin, alpha B |
| Hspa8 | 10.16 | 7.42 | 1142 | 171 | -6.7 | heat shock 70kDa protein 8 |
| **Signaling** | | | | | | |
| Shcbp1 | 7.11 | 9.69 | 138 | 829 | 6.0 | SHC SH2-domain binding protein 1 |
| Adcy7 | 6.96 | 9.25 | 124 | 609 | 5.0 | adenylate cyclase 7 |
| Procr | 10.78 | 9.14 | 1761 | 565 | -3.1 | protein C receptor, endothelial |
| Igfbp4 | 10.46 | 8.81 | 1409 | 449 | -3.2 | insulin-like growth factor binding protein 4 |
| Grb14 | 9.52 | 7.82 | 734 | 225 | -3.2 | growth factor receptor-bound protein 14 |
| Snx12 | 10.85 | 9.01 | 1849 | 517 | -3.6 | sorting nexin 12 |
| Ramp3 | 11.55 | 9.30 | 3007 | 629 | -2.8 | receptor (G protein-coupled) activity modifying protein 3 |
| Appl2 | 11.20 | 8.67 | 2352 | 407 | -5.9 | adaptor protein, phosphotyrosine interaction, PH domain and leucine zipper containing 2 |
| Rgs16 | 10.86 | 7.93 | 1859 | 244 | -7.7 | regulator of G-protein signaling 16 |
| **Transcription/Translation** | | | | | | |
| Krcc1 | 7.45 | 9.56 | 175 | 757 | 4.4 | lysine-rich coiled-coil 1 |
| Srsf1 | 8.41 | 10.40 | 341 | 1350 | 4.0 | serine/arginine-rich splicing factor 1 |
| Gatad2a | 6.29 | 8.27 | 78 | 309 | 3.9 | GATA zinc finger domain containing 2A |
| Srsf2 | 9.85 | 11.53 | 920 | 2962 | 3.2 | serine/arginine-rich splicing factor 2 |
| Hnrnpd | 9.74 | 11.38 | 858 | 2674 | 3.1 | heterogeneous nuclear ribonucleoprotein D (AU-rich element RNA binding protein 1, 37kDa) |
| Neat1 | 8.59 | 10.21 | 387 | 1186 | 3.1 | nuclear paraspeckle assembly transcript 1 (non-protein coding) |
| Lmnb1 | 7.32 | 8.91 | 160 | 480 | 3.0 | laminin B1 |
| Malat1 | 10.59 | 8.91 | 1543 | 483 | -3.2 | metastasis associated lung adenocarcinoma transcript 1 (non-protein coding) |
| Taf1d | 9.14 | 7.36 | 566 | 164 | -3.4 | TATA box binding protein (TBP)-associated factor, RNA polymerase I, D, 41kDa |
| Hnrpdl | 12.03 | 10.23 | 4197 | 1203 | -3.4 | heterogeneous nuclear ribonucleoprotein D-like |
| Rpl41 | 8.37 | 6.56 | 330 | 94 | -3.6 | ribosomal protein L41 |
| Luc7l | 10.50 | 8.64 | 1448 | 398 | -3.6 | LUC7-like (S. cerevisiae) |
| Ccnl1 | 11.77 | 9.85 | 3500 | 922 | -3.8 | cyclin L1 |
| Clk1 | 11.64 | 9.69 | 3199 | 825 | -3.8 | CDC-like kinase 1 |
| Rps10 | 8.29 | 6.29 | 314 | 79 | -4.0 | ribosomal protein S10 |
| Pabpc1 | 9.92 | 7.88 | 971 | 236 | -4.2 | poly(A) binding protein, cytoplasmic 1 |
| Luc7l2 | 11.64 | 9.30 | 3185 | 632 | -5.0 | LUC7-like 2 (S. cerevisiae) |
| Txnip | 10.11 | 7.41 | 1105 | 171 | -7.1 | thioredoxin interacting protein |
| **Transport** | | | | | | |
| Chac2 | 6.77 | 8.65 | 109 | 403 | 3.7 | ChaC, cation transport regulator homolog 2 (E. coli) |
| Slc22a4 | 9.90 | 8.25 | 955 | 304 | -3.1 | solute carrier family 22 (organic cation/zwitterion transporter), member 4 |
| Slc35a2 | 8.95 | 7.28 | 493 | 156 | -3.1 | solute carrier family 35 (UDP-galactose transporter), member A2 |
| Nptx1 | 9.53 | 7.84 | 737 | 229 | -3.2 | neuronal pentraxin I |
| Slc25a37 | 10.84 | 9.14 | 1830 | 565 | -3.2 | solute carrier family 25 (mitochondrial iron transporter), member 37 |

**Supplementary Table 3. Gene expression in EO771/shCxcl1.** Shown are ≥3.0-fold changes in gene expression with a raw score ≥300 in EO771/shCxcl1 or EO771/Scr cells.

| | log 2 | | Raw Score | | FC | |
|---|---|---|---|---|---|---|
| **Function** | **Scr** | **shCxcl1** | **Scr** | **shCxcl1** | **sh/Scr** | **Gene Name** |
| **Adhesion/ECM** | | | | | | |
| Plet1 | 9.07 | 7.15 | 538 | 142 | -3.8 | Placenta Expressed Transcript 1, ECM, wound repair |
| **Apoptosis** | | | | | | |
| CD82 | 11.42 | 9.78 | 2747 | 878 | -3.1 | Kangai 1 (Suppression Of Tumorigenicity 6, Prostate |
| **Immune/Inflammation** | | | | | | |
| Ly6a | 12.93 | 11.29 | 7787 | 2512 | -3.1 | Lymphocyte antigen 6 complex, locus A, Stem cell antigen-1, Sca-1 |
| Il23a | 8.84 | 7.06 | 459 | 133 | -3.5 | Innate and adaptive immunity, autoimmunity, inflammatory cytokines |
| C3 | 8.48 | 6.55 | 358 | 94 | -3.8 | Complement Component 3, innate and adaptive immunity, inflammation |
| Cxcl1 | 11.06 | 9.07 | 2137 | 536 | -4.0 | Chemokine (C-X-C Motif) Ligand 1 (Melanoma Growth Stimulating Activity, Alpha) |
| CD68 | 8.62 | 6.54 | 394 | 93 | -4.2 | Macrophage phagocytosis |
| **Invasion/Motility** | | | | | | |
| Plau | 9.89 | 7.05 | 949 | 133 | -7.3 | Plasminogen activator urokinase |
| **Mitosis** | | | | | | |
| Clip4 | 8.61 | 6.91 | 391 | 120 | -3.6 | CAP-GLY Domain Containing Linker Protein Family, Member 4, centrosome |
| Piwil2 | 9.46 | 7.64 | 707 | 199 | -3.6 | Piwi-Like RNA-Mediated Gene Silencing 2, meiosis, oncogene when overexpressed |
| **Signaling** | | | | | | |
| Rgs16 | 10.79 | 9.15 | 1771 | 568 | -3.2 | Regulator of G-Protein Signaling 16 |
| **Transport** | | | | | | |
| Slc7a3 | 7.76 | 9.39 | 217 | 673 | 3.1 | Solute Carrier Family 7 (Cationic Amino Acid Transporter, Y+ System), Member 3 |

**Supplementary Table 4. Gene expression in EO771 isografts from SB25002-treated mice.** Shown are genes with ≥3-fold change in expression and a raw score ≥300 in mammary tumors from mice treated with vechle or 20 mg/kg SB25002.

| | **log2** | | **Raw Score** | | **FC** | |
|---|---|---|---|---|---|---|
| **Function** | **Vehicle** | **SB25002** | **Vehicle** | **SB25002** | **SB/Vehicle** | **Gene Name** |
| **Adhesion/ECM** | | | | | | |
| Cgref1 | 11.6121 | 13.3081 | 3130 | 10142 | 3.2 | cell growth regulator with EF hand domain 1 |
| Nrcam | 8.0089 | 9.6418 | 258 | 799 | 3.1 | neuronal cell adhesion molecule |
| **Apoptosis** | | | | | | |
| Cidea | 8.7618 | 7.1531 | 434 | 142 | -3.0 | cell death-inducing DNA fragmentation factor, alpha subunit-like effector A |
| **Differentiation** | | | | | | |
| Krt18 | 13.0294 | 14.6218 | 8360 | 25211 | 3.0 | keratin 18 |
| Lor | 9.5320 | 6.3053 | 740 | 79 | -9.4 | loricrin |
| **Immune/Inflammation** | | | | | | |
| Ly6c1 | 14.9712 | 13.4000 | 32121 | 10809 | -3.0 | lymphocyte antigen 6 complex, locus C1 |
| Ly6d | 8.8947 | 7.3091 | 476 | 159 | -3.0 | lymphocyte antigen 6 complex, locus D |
| Tmem176a | 10.6826 | 9.0431 | 1644 | 528 | -3.1 | transmembrane protein 176A |
| C1qtnf1 | 10.4737 | 8.6785 | 1422 | 410 | -3.5 | C1q and tumor necrosis factor related protein 1 |
| Tmem176b | 15.2836 | 13.2279 | 39886 | 9594 | -4.2 | transmembrane protein 176B |
| **Invasion/Motility** | | | | | | |
| Tspan2 | 9.2743 | 10.8752 | 619 | 1878 | 3.0 | tetraspanin 2 |
| Tnnt2 | 9.8600 | 11.4300 | 929 | 2759 | 3.0 | troponin T2, cardiac |
| Serpinb1a | 12.8849 | 10.8336 | 7564 | 1825 | -4.1 | serine (or cysteine) peptidase inhibitor, clade B, member 1a |
| **Metabolism** | | | | | | |
| Tex101 | 8.5414 | 10.3233 | 373 | 1281 | 3.4 | testis expressed gene 101 |
| Cpt1c | 6.8928 | 8.5777 | 119 | 382 | 3.2 | carnitine palmitoyltransferase 1c |
| Pla2g5 | 12.3834 | 10.8200 | 5343 | 1808 | 3.0 | phospholipase A2, group V |
| Gsta2 | 8.9964 | 7.1455 | 511 | 142 | -3.6 | glutathione S-transferase, alpha 2 (Yc2) |
| Plin4 | 10.3196 | 7.7437 | 1278 | 214 | -6.0 | perilipin 4 |
| Gpd1 | 9.8930 | 7.1120 | 951 | 138 | -6.9 | glycerol-3-phosphate dehydrogenase 1 (soluble) |
| Hp | 14.7010 | 11.5504 | 26634 | 2999 | -8.9 | haptoglobin |
| **Proliferation** | | | | | | |
| Figf | 10.8829 | 9.3714 | 1888 | 640 | 3.0 | c-fos induced growth factor |
| Greb1 | 12.9176 | 14.7121 | 7737 | 26840 | 3.5 | gene regulated by estrogen in breast cancer protein |
| **Signaling** | | | | | | |
| F3 | 9.0419 | 10.6820 | 527 | 1643 | 3.1 | coagulation factor III |
| P2rx3 | 9.2673 | 7.6281 | 616 | 198 | -3.1 | purinergic receptor P2X, ligand-gated ion channel, 3 |
| Sncg | 11.6458 | 9.5825 | 3204 | 767 | -4.2 | synuclein, gamma |
| **Transcription/Translation** | | | | | | |
| Anp32a | 12.4230 | 14.0442 | 5491 | 16894 | 3.1 | acidic (leucine-rich) nuclear phosphoprotein 32 family, member A |
| Pcbd1 | 11.4736 | 9.7331 | 2844 | 851 | -3.3 | pterin 4 alpha carbinolamine dehydratase/dimerization cofactor of hepatocyte nuclear factor 1 alpha (TCF1) 1 |
| Glod5 | 9.8789 | 7.7521 | 942 | 216 | -4.4 | glyoxalase domain containing 5 |
| **Transport** | | | | | | |
| Scara5 | 11.5306 | 9.7621 | 2958 | 868 | -3.4 | scavenger receptor class A, member 5 (putative) |
| Mup-ps16 | 9.3871 | 7.5667 | 670 | 190 | -3.5 | major urinary protein, pseudogene 16 |
| Mup4 | 10.2305 | 8.1747 | 1201 | 289 | -4.2 | major urinary protein 4 |
| Retn | 9.6222 | 7.5379 | 788 | 186 | -4.2 | resistin |
| Mup19 | 11.7530 | 8.9928 | 3451 | 509 | -6.8 | major urinary protein 19 |

**Supplemental Table 5. Gene expression in MC/Plac1 cells.** Shown are genes with ≥3-fold change in expression with a raw score ≥300 in MC/Ctl or MC/Plac1 cells.

| | log2 | | Raw Score | | FC | |
|---|---|---|---|---|---|---|
| **Function** | **MC/Ctl** | **MC/Plac1** | **MC/Ctl** | **MC/Plac1** | **Plac1/Ctl** | **Gene Name** |
| **Adhesion/ECM** | | | | | | |
| Cldn1 | 8.07 | 10.78 | 269 | 1758 | 6.5 | Claudin 1 |
| Hs6st2 | 6.51 | 8.59 | 91 | 385 | 4.2 | Heparan Sulfate 6-O-Sulfotransferase 2 |
| Vcan | 6.72 | 8.63 | 105 | 396 | 3.8 | Versican |
| Lgals9 | 7.32 | 9.22 | 160 | 596 | 3.7 | Lectin, Galactoside-Binding, Soluble, 9 |
| Fn1 | 8.56 | 6.93 | 377 | 122 | -3.1 | Fibronectin 1 |
| Plec | 10.97 | 9.34 | 2006 | 648 | -3.1 | Plectin |
| Matn2 | 8.44 | 6.66 | 347 | 101 | -3.4 | Matrilin 2 |
| Emp2 | 11.52 | 9.73 | 2937 | 849 | -3.4 | Epithelial membrane protein 2 |
| Lrrc8a | 8.5 | 6.54 | 362 | 93 | -3.9 | Leucine Rich Repeat Containing 8 Family, Member A |
| **Differentiation** | | | | | | |
| Krt16 | 6.12 | 10.4 | 70 | 1351 | 19.4 | Keratin 16 |
| Fam198b | 7.26 | 9.21 | 153 | 592 | 3.9 | Family With Sequence Similarity 198, Member B |
| Sema3c | 7.96 | 9.85 | 249 | 923 | 3.7 | Sema Domain, Immunoglobulin Domain (Ig), Short Basic Domain, Secreted |
| Pard6g | 8.22 | 9.89 | 298 | 949 | 3.2 | Par-6 Family Cell Polarity Regulator Gamma |
| Scara5 | 9.28 | 7.64 | 622 | 199 | -3.1 | Scavenger Receptor Class A, Member 5 |
| Cwh43 | 8.98 | 7.12 | 505 | 139 | -3.6 | Cell Wall Biogenesis 43 C-Terminal Homolog |
| **Immune/Inflammation** | | | | | | |
| Cxcl11 | 5.25 | 10.46 | 38 | 1409 | 37.0 | Chemokine (C-X-C Motif) Ligand 11 |
| Iigp1 | 7.05 | 11.43 | 133 | 2759 | 21.0 | Interferon inducible GTPase 1 |
| Tgtp1 | 8.95 | 13.22 | 495 | 9541 | 19.3 | T-cell specific GTPase 1 |
| Cxcl10 | 7.27 | 10.98 | 154 | 2020 | 13.1 | Chemokine (C-X-C Motif) Ligand 10 |
| Ifi27l2a | 7.4 | 11.03 | 169 | 2091 | 12.4 | Interferon, Alpha-Inducible Protein 27-Like 2 |
| Irg1 | 6.19 | 9.74 | 73 | 855 | 11.7 | Immunoresponsive 1 Homolog (Mouse) |
| Trim30a | 8.62 | 11.99 | 393 | 4068 | 10.5 | Tripartite Motif Containing 5 |
| Gbp6 | 7.9 | 11.24 | 239 | 2419 | 10.1 | Guanylate Binding Protein Family, Member 6 |
| Ifit3 | 9.91 | 13.22 | 962 | 9541 | 9.9 | Interferon-Induced Protein With Tetratricopeptide Repeats 3 |
| Zbp1 | 7.68 | 10.8 | 205 | 1783 | 8.8 | Z-DNA Binding Protein 1 |
| Rsad2 | 9.62 | 12.7 | 787 | 6654 | 8.6 | Radical S-Adenosyl Methionine Domain Containing 2 |
| Ifit1 | 10.63 | 13.62 | 1585 | 12590 | 7.9 | Interferon-Induced Protein With Tetratricopeptide Repeats 1 |
| Gbp3 | 10.39 | 13.22 | 1342 | 9541 | 7.1 | Guanylate Binding Protein 3 |
| Ccl5 | 10.87 | 13.69 | 1872 | 13216 | 7.1 | Chemokine (C-C Motif) Ligand 5 |
| Ddx60 | 8.53 | 11.35 | 370 | 2610 | 7.1 | DEAD (Asp-Glu-Ala-Asp) Box Polypeptide 60 |
| Irf7 | 8.59 | 11.4 | 385 | 2702 | 7.0 | Interferon Regulatory Factor 7 |
| Il33 | 6.21 | 8.64 | 74 | 399 | 5.4 | Interleukin-33 |
| Oasl2 | 9.92 | 12.29 | 969 | 5008 | 5.2 | 2'-5'-Oligoadenylate Synthetase-Like |
| H2-Q7 | 6.63 | 8.94 | 99 | 491 | 5.0 | Histocompatibility 2, Q region locus 7 |
| Ifit2 | 10.32 | 12.6 | 1278 | 6208 | 4.9 | Interferon-Induced Protein With Tetratricopeptide Repeats 2 |
| Ifi44 | 10.03 | 12.28 | 1046 | 4973 | 4.8 | Interferon-Induced Protein 44 |
| Ccl2 | 7.52 | 9.75 | 184 | 861 | 4.7 | Chemokine (C-C Motif) Ligand 2 |
| Usp18 | 10.98 | 13.2 | 2020 | 9410 | 4.7 | Ubiquitin Specific Peptidase 18 |
| Isg15 | 11.38 | 13.56 | 2665 | 12077 | 4.5 | ISG15 Ubiquitin-Like Modifier |
| Dhx58 | 7.63 | 9.73 | 198 | 849 | 4.3 | DEXH (Asp-Glu-X-His) Box Polypeptide 58 |
| Igtp | 10.94 | 12.95 | 1965 | 7913 | 4.0 | Interferon gamma induced GTPase |
| Gbp2 | 11.13 | 12.98 | 2241 | 8079 | 3.6 | Guanylate Binding Protein 2, Interferon-Inducible |
| Ifih1 | 10.17 | 11.94 | 1152 | 3929 | 3.4 | Interferon Induced With Helicase C Domain 1 |
| Ifi47 | 10.92 | 12.69 | 1938 | 6608 | 3.4 | Interferon gamma inducible protein 47 |
| Irgm1 | 11.47 | 13.2 | 2837 | 9410 | 3.3 | Immunity-related GTPase |
| Lrr1 | 7.12 | 8.83 | 139 | 455 | 3.3 | Leucine Rich Repeat Protein 1 |
| Cd274 | 7.35 | 8.94 | 163 | 491 | 3.0 | Programmed Cell Death 1 Ligand 1 |
| Oasl1 | 8.03 | 9.69 | 261 | 826 | 3.2 | 2'-5'-Oligoadenylate Synthetase-Like1 |
| Gbp7 | 9.52 | 11.17 | 734 | 2304 | 3.1 | Guanylate Binding Protein 7 |
| Aebp1 | 9.79 | 11.43 | 885 | 2759 | 3.1 | Adipocyte Enhancer Binding Protein 1 |
| Oas1a | 11.21 | 12.81 | 2369 | 7181 | 3.0 | 2'-5'-Oligoadenylate Synthetase 1, 44/46kDa |
| Cxcl2 | 9.38 | 7.69 | 666 | 207 | -3.2 | Chemokine (C-X-C Motif) Ligand 2 |
| Cd68 | 10.49 | 8.48 | 1438 | 357 | -4.0 | Scavenger Receptor Class D, Member 1 |
| Il1a | 8.44 | 5.93 | 347 | 61 | -5.7 | Interleukin 1, Alpha |
| **Invasion/Motility** | | | | | | |
| Fhod3 | 5.32 | 9.92 | 40 | 969 | 24.3 | Formin Homology 2 Domain Containing 3 |
| Serpinb2 | 5.20 | 9.30 | 37 | 630 | 17.1 | Serpin Peptidase Inhibitor, Clade B (Ovalbumin), Member 2 |
| Plat | 7.68 | 9.68 | 205 | 820 | 4.0 | Plasminogen Activator, Tissue |
| Pfn2 | 7.82 | 9.77 | 226 | 873 | 3.9 | Profilin 2 |
| Myo10 | 9.93 | 11.63 | 976 | 3169 | 3.3 | myosin X |
| Psmb9 | 9.68 | 11.42 | 820 | 2740 | 3.3 | Proteasome (Prosome, Macropain) Subunit, Beta Type, 9 |
| Hmmr | 7.89 | 9.54 | 237 | 744 | 3.2 | Hyaluronan-Mediated Motility Receptor |
| Malat1 | 12.05 | 10.16 | 4240 | 1144 | -4.0 | Metastasis Associated Lung Adenocarcinoma Transcript 1 |
| Tnfaip2 | 10.8 | 8.69 | 1783 | 413 | -4.3 | Tumor Necrosis Factor, Alpha-Induced Protein 2 |
| Mmp3 | 9.06 | 6.9 | 534 | 119 | -4.5 | Matrix Metallopeptidase 3 (Stromelysin 1) |
| Ctsc | 8.72 | 6.48 | 422 | 89 | -4.7 | Cathepsin C |
| Mmp10 | 10.39 | 7.88 | 1342 | 236 | -5.7 | Matrix Metallopeptidase 10 (Stromelysin 2) |
| **Metabolism** | | | | | | |
| Cmpk2 | 7.56 | 10.03 | 189 | 1046 | 5.9 | CMP (UMP-CMP) Kinase 2, Mitochondrial |
| Cyp1b1 | 7.84 | 9.98 | 229 | 1010 | 4.6 | Cytochrome P450, Family 1, Subfamily B, Polypeptide 11 |
| Ugt1a1 | 9.91 | 11.59 | 962 | 3083 | 3.2 | UDP Glucuronosyltransferase 1 Family, Polypeptide A1 |
| Ckb | 10.01 | 8.41 | 1031 | 340 | -3.0 | Creatine kinase, brain |
| Pck2 | 10.36 | 8.67 | 1314 | 407 | -3.2 | Phosphoenolpyruvate Carboxykinase 2 |
| Shmt2 | 10.48 | 8.85 | 1428 | 461 | -3.1 | Serine Hydroxymethyltransferase 2 (Mitochondrial) |
| Mthfd2 | 11.21 | 9.48 | 2369 | 714 | -3.2 | Methylenetetrahydrofolate Dehydrogenase (NADP+ Dependent) 2 |

| | | | | | | |
|---|---|---|---|---|---|---|
| Psph | 12.25 | 10.51 | 4871 | 1458 | -3.4 | Phosphoserine Phosphatase1 |
| Aldoc | 10.29 | 8.63 | 1252 | 396 | -3.6 | Aldolase C, Fructose-Bisphosphate |
| Stbd1 | 11.22 | 9.37 | 2385 | 662 | -3.6 | Starch Binding Domain 1 |
| Ero1l | 11.6 | 9.65 | 3104 | 803 | -3.7 | Endoplasmic Oxidoreductin-1-Like Protein |
| Aspa | 10.03 | 8.07 | 1046 | 269 | -3.9 | Aspartoacylase |
| Acot2 | 8.52 | 6.52 | 367 | 92 | -4.0 | Acyl-CoA Thioesterase 2 |
| Gpt2 | 10.54 | 10.22 | 1489 | 1193 | -4.0 | Glutamic Pyruvate Transaminase |
| Pycr1 | 9.5 | 7.49 | 724 | 180 | -4.0 | Pyrroline-5-Carboxylate Reductase 1 |
| Hmox1 | 12.11 | 10.07 | 4421 | 1075 | -4.1 | Heme Oxygenase (Decycling) 1 |
| Acsbg1 | 8.56 | 6.44 | 377 | 87 | -4.3 | Acyl-CoA Synthetase Bubblegum Family Member 1 |
| Cth | 9.87 | 7.55 | 936 | 187 | -5.0 | Cystathionine Gamma-Lyase |
| Gsta1/Gsta2 | 11.5 | 7.27 | 2896 | 154 | -16.7 | Glutathione S-Transferase Alpha 1/Alpha 2 |
| Inmt | 11.59 | 6.63 | 3083 | 99 | -31.3 | Indolethylamine N-Methyltransferase |
| **Mitosis** | | | | | | |
| Kif20a | 8.35 | 10.35 | 326 | 1305 | 4.0 | Kinesin Family Member 20A1 |
| Nusap1 | 8.07 | 10.06 | 269 | 1067 | 4.0 | Nucleolar And Spindle Associated Protein 1 |
| Plk1 | 7.52 | 9.35 | 184 | 653 | 3.6 | Polo-like Kinase 1 |
| Ttk | 7.18 | 9.01 | 145 | 516 | 3.6 | TTK Protein Kinase |
| Kif23 | 6.93 | 8.7 | 122 | 416 | 3.4 | Kinesin Family Member 23 |
| Prc1 | 8.62 | 10.36 | 393 | 1314 | 3.4 | Protein Regulator Of Cytokinesis 1 |
| Aurkb | 7.83 | 9.55 | 228 | 750 | 3.3 | Aurora B Kinase |
| Kif2c | 8.09 | 9.8 | 272 | 891 | 3.3 | Kinesin Family Member 2c |
| Aspm | 7.57 | 9.25 | 190 | 609 | 3.2 | Asp (Abnormal Spindle) Homolog, Microcephaly Associated |
| Zwilch | 8.05 | 9.73 | 265 | 849 | 3.2 | Zwilch Kinetochore Protein |
| Ncapg | 7.26 | 8.92 | 153 | 484 | 3.2 | Non-SMC Condensin I Complex, Subunit G |
| Birc5 | 9.69 | 11.34 | 826 | 2592 | 3.1 | Baculoviral IAP Repeat Containing 5 |
| Mastl | 6.62 | 8.26 | 98 | 307 | 3.1 | Microtubule Associated Serine/Threonine Kinase-Like |
| Kif22 | 7.1 | 8.72 | 137 | 422 | 3.1 | Kinesin Family Member 22 |
| Nuf2 | 7.54 | 9.17 | 186 | 576 | 3.1 | NUF2, NDC80 Kinetochore Complex Component |
| Cenpe | 8.28 | 9.87 | 311 | 936 | 3.0 | Centromere protein E |
| Fam110c | 9.45 | 7.83 | 699 | 228 | -3.1 | Family With Sequence Similarity 110, Member C |
| **Proliferation** | | | | | | |
| Plac1 | 7.51 | 11.37 | 182 | 2647 | 14.5 | Placental-specific 1 |
| Shcbp1 | 8.21 | 10.16 | 296 | 1144 | 3.9 | SHC SH2-Domain Binding Protein 11 |
| Wnt4 | 8.42 | 10.2 | 343 | 1176 | 3.4 | Wingless-Type MMTV Integration Site Family, Member 4 |
| Rab32 | 8.22 | 10.09 | 298 | 1090 | 3.7 | RAB32, Member RAS Oncogene Family |
| Afp | 6.4 | 8.23 | 84 | 300 | 3.6 | Alpha-fetoprotein |
| Mertk | 6.55 | 8.29 | 94 | 313 | 3.3 | C-Mer Proto-Oncogene Tyrosine Kinase |
| Ccna2 | 8.84 | 10.53 | 458 | 1479 | 3.2 | Cyclin A2 |
| Smpdl3b | 8.33 | 9.97 | 322 | 1003 | 3.1 | Sphingomyelin Phosphodiesterase, Acid-Like 3B1 |
| Ghr | 9.88 | 11.52 | 942 | 2937 | 3.1 | Growth hormone receptor |
| Bnip3 | 11.61 | 9.96 | 3126 | 996 | -3.1 | BCL2/Adenovirus E1B 19kDa Interacting Protein 3 |
| Trib3 | 11.13 | 9.25 | 2241 | 609 | -3.6 | Tribbles Pseudokinase 3 |
| Gas5 | 9.82 | 7.91 | 904 | 241 | -3.8 | Growth Arrest-Specific 5 |
| Phlda2 | 9.71 | 7.75 | 838 | 215 | -3.9 | Pleckstrin Homology-Like Domain, Family A, Member 2 |
| Vegfa | 11.76 | 9.67 | 3468 | 815 | -4.2 | Vascular endothelial growth factor A |
| Areg | 11.03 | 8.94 | 2091 | 491 | -4.3 | Amphiregulin |
| Reep6 | 10.85 | 8.69 | 1846 | 413 | -4.5 | Receptor Accessory Protein 6 |
| Csf1r | 9.98 | 7.78 | 1010 | 220 | -4.6 | Colony Stimulating Factor 1 Receptor |
| Prkg2 | 11.34 | 9.11 | 2592 | 553 | -4.7 | Protein Kinase, CGMP-Dependent, Type II |
| Artn | 9.45 | 7.15 | 699 | 142 | -4.9 | Artemin |
| Ndrg1 | 11.85 | 9.48 | 3692 | 714 | -5.0 | N-Myc Downstream Regulated 1 |
| Chac1 | 11.53 | 9.02 | 2957 | 519 | -5.7 | ChaC, Cation Transport Regulator Homolog 1 |
| Fgf21 | 8.46 | 5.95 | 352 | 62 | -5.7 | Fibroblast growth factor 21 |
| Fam129a | 9.37 | 6.76 | 662 | 108 | -6.1 | Family With Sequence Similarity 129, Member A |
| **Secretion** | | | | | | |
| Inhbb | 8.76 | 11.76 | 434 | 3468 | 8.0 | Activin Beta-B Chain |
| Fst | 7.18 | 9.81 | 145 | 898 | 6.2 | Follistatin |
| Tac1 | 7.03 | 8.91 | 131 | 481 | 3.7 | Tachykinin, Precursor 1 |
| **Transport** | | | | | | |
| Rtp4 | 7.03 | 8.84 | 131 | 458 | 3.5 | Receptor (Chemosensory) Transporter Protein |
| Abca1 | 8.81 | 10.5 | 449 | 1448 | 3.2 | ATP-Binding Cassette, Sub-Family A (ABC1), Member 1 |
| Slc1a4 | 9.29 | 7.59 | 626 | 193 | -3.2 | Solute Carrier Family 1 (Glutamate/Neutral Amino Acid Transporter), Member 4 |
| Fxyd2 | 10.25 | 8.26 | 1218 | 307 | -4.0 | FXYD Domain Containing Ion Transport Regulator 2 |
| Slc7a11 | 8.84 | 5.84 | 458 | 57 | -8.0 | Solute carrier family 7 (anionic amino acid transporter light chain, xc-system), member 11 |
| **Transcription/Translation** | | | | | | |
| Hist1h2b | 8.77 | 12.55 | 437 | 5997 | 13.9 | Histone cluster 1, H2b |
| Csprs | 5.81 | 8.28 | 56 | 311 | 5.9 | Component of Sp100-rs |
| Gadd45g | 9.95 | 12.36 | 989 | 5257 | 5.3 | Growth Arrest And DNA-Damage-Inducible, Gamma |
| Hist1h3b | 6.22 | 8.58 | 75 | 383 | 5.1 | Histone cluster 1, H3b |
| Hist1h4a | 6.16 | 8.3 | 72 | 315 | 4.6 | Histone cluster 1, H4a |
| Hopx | 7.44 | 9.53 | 174 | 739 | 4.3 | HOP Homeobox |
| Depdc1a | 7.35 | 9.26 | 163 | 613 | 3.8 | DEP Domain Containing 1 |
| Foxm1 | 6.6 | 8.45 | 97 | 350 | 3.6 | Forkhead Box M1 |
| Hist2h2aa | 8.44 | 10.23 | 347 | 1201 | 3.5 | Histone cluster 2, H2aa |
| Nr4a2 | 7.12 | 8.92 | 139 | 484 | 3.5 | Nuclear Receptor Subfamily 4, Group A, Member 2 |
| Stat1 | 9.86 | 11.59 | 929 | 3083 | 3.3 | Signal transducer and activator of transcription 1, 91kDa |
| Atxn7l1 | 7.07 | 8.8 | 134 | 446 | 3.3 | Ataxin 7-Like 1 |
| Id3 | 9.42 | 11.09 | 685 | 2180 | 3.2 | Inhibitor of DNA binding 3, dominant negative helix-loop-helix protein |
| Sp100 | 7.39 | 9.02 | 168 | 519 | 3.1 | SP100 Nuclear Antigen |
| Txnip | 10.35 | 8.65 | 1305 | 402 | -3.2 | Thioredoxin Interacting Protein |
| Rpl30 | 8.36 | 6.51 | 329 | 91 | -3.6 | Ribosomal Protein L30 |

**Supplementary Table 6. List of primers for qRT-PCR analysis**

| Gene Accesion # | Primer Name | Sequence | Ampliconsize (bp) | Primer Location |
|---|---|---|---|---|
| Cxcl1 | | | | |
| NM_008176.3 | mCxcl1 rRNA-F | GTGTCTAGTTGGTAGGGCATAAT | 94 | 823-846 |
| | mCxcl1 rRNA-R | CAGTCCTTTGAACGTCTCTGT | | 896-917 |
| Ccl2 | mCcl2 rRNA-F | CTCGGACTGTGATGCCTTAAT | 106 | 538-559 |
| NM_011333.3 | mCcl2 rRNA-R | TGGATCCACACCTTGCATTTA | | 623-644 |
| Ccl5 | mCcl5 rRNA-F | CCAGAGAAGAAGTGGGTTCAAG | 100 | 283-305 |
| NM_013653.3 | mCcl5 rRNA-R | AGCAATGACAGGGAAGCTATAC | | 361-383 |
| Ccl7 | mCcl7 rRNA-F | GGATAGGAGCTGTCTGTAGGA | 128 | 472-493 |
| NM_013654.3 | mCcl7 rRNA-R | CATGAGGTCTCCAGAGCTTTAC | | 578-600 |
| CD274 | mCD274 rRNA-F | GAGTGCAGATTCCCTGTAGAAC | 99 | 198-220 |
| NM_021893.3 | mCD274 rRNA-R | CTCCTCTCCTGCCACAAAC | | 227-297 |
| Lif | mLif rRNA-F | CGCCAATGCTCTCTTCATTTC | 98 | 177-198 |
| NM_008501.2 | mLif rRNA-R | ATGGGAAGTCTGTCATGTTAGG | | 253-275 |
| Plac1 | mPlac1 rRNA-F | GAGGTCCTTCTTGTGTAGTCATC | 96 | 790-813 |
| NM_019538.4 | mPlac1 rRNA-R | ATCTGGGCACTATATGGGTTTC | | 864-886 |
| 18S rRNA | m18S rRNA-F | TCGGAACTGAGGCCATGATT | 146 | 900-919 |
| NR_003278 | m18S rRNA-R | CCTCCGACTTTCGTTCTTGATT | | 1024-1045 |
| CD68 | mCD68 rRNA-F | CCCACCTGTCTCTCTCATTTC | 107 | 545-566 |
| NM_001291058.1 | mCD68 rRNA-R | GTATTCCACCGCCATGTAGT | | 632-652 |
| Ly6a | mLy6a rRNA-F | CTCAGGAGGCAGCAGTTATT | 106 | 164-184 |
| NM_001271416.1 | mLy68 rRNA-R | GTACCCAGGATCTCCATACTTTC | | 247-270 |
| IL23a | mIL23a rRNA-F | CCAGCGGGACATATGAATCTAC | 95 | 148-170 |
| NM_031252.2 | mIL23a rRNA-R | TGTGGGTCACAACCATCTTC | | 223-243 |
| Plau | mPlau rRNA-F | GATTCTGGAGGACCGCTTATC | 105 | 1129-1150 |
| NM_008873.3 | mPlau rRNA-R | GTAGACACCGGGCTTGTTT | | 1215-1234 |
